# Sonochemical Synthesis of 2’-Hydroxy-Chalcone Derivatives with Potential Anti-Oomycete Activity

**DOI:** 10.3390/antibiotics9090576

**Published:** 2020-09-04

**Authors:** Génesis López, Marco Mellado, Enrique Werner, Bastián Said, Patricio Godoy, Nelson Caro, Ximena Besoain, Iván Montenegro, Alejandro Madrid

**Affiliations:** 1 Laboratorio de Productos Naturales y Síntesis Orgánica (LPNSO), Departamento de Química, Facultad de Ciencias Naturales y Exactas, Universidad de Playa Ancha, Avda. Leopoldo Carvallo 270, Playa Ancha, Valparaíso 2340000, Chile; genesis.lopez.miranda@gmail.com; 2 Instituto de Química, Facultad de Ciencias, Pontificia Universidad Católica de Valparaíso, Av. Universidad #330, Curauma, Valparaíso 2340000, Chile; marco.mellado@pucv.cl; 3 Departamento de Ciencias Básicas, Campus Fernando May Universidad del Biobío. Avda. Andrés Bello 720, Casilla 447, Chillán 3780000, Chile; ewerner@ubiobio.cl; 4 Departamento de Química, Universidad Técnica Federico Santa María, Av. Santa María 6400, Vitacura, Santiago 7630000, Chile; bastian.said@usm.cl; 5 Instituto de Microbiología Clínica, Facultad de Medicina, Universidad Austral de Chile, Los Laureles s/n, Isla Teja, Valdivia 5090000, Chile; patricio.godoy@uach.cl; 6 Centro de Investigación Australbiotech, Universidad Santo Tomás, Avda. Ejército 146, Santiago 8320000, Chile; ncaro@australbiotech.cl; 7 Escuela de Agronomía Pontificia Universidad Católica de Valparaíso, Quillota, SanFrancisco s/n La Palma, Quillota 2260000, Chile; ximena.besoain@pucv.cl; 8 Escuela de Obstetricia y Puericultura, Facultad de Medicina, Universidad de Valparaíso, Angamos 655, Reñaca, Viña del Mar 2520000, Chile

**Keywords:** chalcone, ultrasound, *Phytophthora infestans*

## Abstract

This work reports on the synthesis of eight new 2′-hydroxy-chalcones with potential anti-phytopathogenic applications in agroindustry, AMONG others, via Claisen–Schmidt condensation and ultrasound assisted reaction. Assays showed three chalcones with allyl moieties strongly inhibited growth of phytopathogenic oomycete *Phytophthora infestans*; moreover, compound **8a** had a half maximal effective concentration (EC_50_) value (32.5 µg/mL) similar to that of metalaxyl (28.6 µg/mL). A software-aided quantitative structure–activity relationship (QSAR) analysis of the whole series suggests that the structural features of these new chalcones—namely, the fluoride, hydroxyl, and amine groups over the carbon 3′ of the chalcone skeleton—increase anti-oomycete activity.

## 1. Introduction

In recent years, worldwide per capita food consumption has increased while growth rates in global agricultural production and crop yields have declined [1]; this latter is largely due to phytosanitary problems like pests, fungi, bacteria, and, mainly in the post-harvest phase, diseases produced by oomycetes [2,3,4]. Of the diseases caused by oomycetes, root rot and fruit blight [5] are estimated to render up to 30% of the harvested vegetables and fruits inedible during postharvest handling [6]. As the main causal agent of stem, root, and fruit rot in commercial cultivars, genus *Phytophthora* [7] is present scattered in almost all producing regions worldwide, causing heavy economic losses and disastrous consequences for natural ecosystems [8].

Specifically, oomycete *Phytophthora infestans* is renowned as the most significant pathogen of the genus, and is responsible for the grave potato and tomato disease known as late blight or potato blight [9]. While there are several fungicides presently used as pre-and post-harvest treatments for control of *P. infestans* [10], their use leaves residues on food and in cultivar soils, increases pathogen resistance, and damages human health and the environment [11]. The prevalence of *P. infestans* and the downsides to chemical controls have stimulated modern agriculture research into alternative microorganism control methods. In addressing these shortcomings, natural product derivatives appear to be a viable approach to reducing late blight disease [12].

Chalcones are well documented as biologically active natural products [13], with potential use in diseases caused by *Phytophthora* spp. In this context, several studies have shown that natural and synthetic chalcones with –OH and –*O-*alkyl chains linked to different positions of the A and B rings are known to have bactericidal, antifungal, anthelmintic, antiviral, and anti-oomycetes activities [14,15,16,17,18]. These reports illustrate that antimicrobial activity depends on the number, type, and length of side chain, and position of these substituents in the aromatic ring. Due to the importance of hydroxyl groups and alkyl side chains, this work reports on a facile sonochemical synthesis of a series of 2′-hydroxy-chalcone derivatives, in which oxyalkyl chains were attached to the A aromatic ring in different positions and variable lengths. The anti-oomycete activities of these chalcone derivatives were tested against *P. infestans* to evaluate the effect of the 2′ position of the hydroxyl group as well as the position and length of the alkyl chain.

## 2. Results and Discussion

**Scheme 1:**
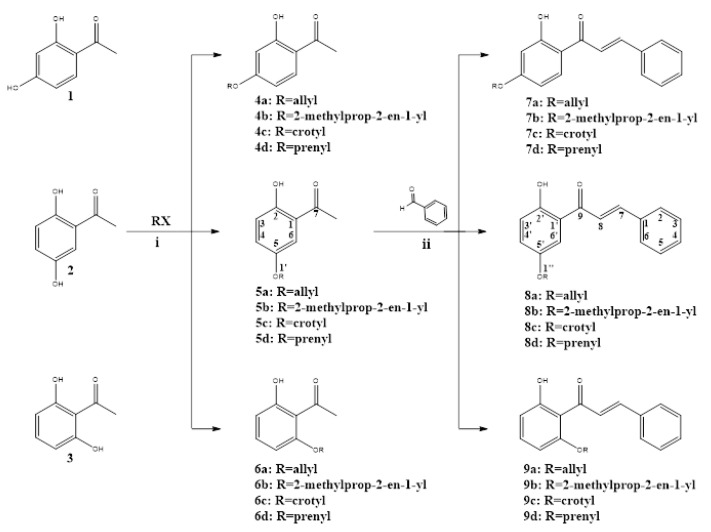
Route for the synthesis of oxyalkylated chalcones. Reagents and conditions: (i) K_2_CO_3_, acetone, ultrasound at 45–50 °C for 20–30 min. (ii) NaOH, ethanol, ultrasound at 30–35 °C for 3 h.

This study synthesized twelve derivatives of 2′-hydroxy-chalcone (**7a**–**d**, **8a**–**d**, and **9a**–**d**), characterized by spectroscopy. The base structure was manipulated to include different oxyalkyl chains—allyl, 2-methyl-3propenyl, crotyl, and prenyl—in the 4′, 5′, and 6′ positions, as shown in Scheme 1.

As shown Scheme 1, alkoxy acetophenone (**4a**–**d**, **5a**–**d**, and **6a**–**d**) synthesis had good yield for the alkylation of the corresponding acetophenone, with the desired alkyl halide in the presence of K_2_CO_3_ under ultrasound irradiation. Alkoxy acetophenones obtained were characterized by nuclear magnetic resonance (NMR) spectra analysis. This stage produced five molecules never before described, which, due mainly to the use of ultrasound irradiation, were synthesized nearly stoichiometric. This was notably true for those derived from 2′,6′-dihydroxyacetophenone, whose double hydrogen bridge, under normal synthesis conditions, is obtained inefficiently [19]. From here, 2′-hydroxy-chalcone derivatives were prepared by Claisen–Schmidt condensation of the different alkoxy acetophenones with benzaldehyde under ultrasound irradiation [20], resulting in a known series (**7a**–**d**) and two new series of molecules (**8a**–**d** and **9a**–**d**). The mild reaction conditions, shorter reaction time, and high yields make this reaction more efficient than the classic reflux agitation method used for chalcone synthesis [21].

Furthermore, synthesized chalcones derivatives (**7a**–**d**, **8a**–**d**, and **9a**–**d**), at purity values over 92%, were evaluated for anti-oomycete activity against *P. infestans*, demonstrating significant mycelial growth inhibition (Table 1).

Furthermore, all compounds tested showed strong anti-oomycete activity and were superior to difenoconazole. Among them, compound **8a** was the most potent derivative of the series, with activity similar to that of metalaxyl.

A membrane damage experiment—based on the direct action of the compounds on the formation of sterol in the cells—was performed to establish the possible death pathway of *P. infestans* strains. Compound effects were compared to 2% sodium dodecyl sulfate (SDS), an anionic surfactant that produces 100% cell lysis. Percentage of membrane lysis of *P. infestans* strains are summarized in Table 2.

This type of test is based on the direct action of the compounds on the formation of sterol in the cells of the oomycete membranes’ paper anterior.

The membrane damage test showed antifungal effectiveness of compound 8a against *P. infestans* similar to a chaotropic agent, like SDS. These results are consistent with previous reports that short oxyalkylated chains significantly enhance and improve antimicrobial activities [22].

Additionally, some structure–activity relationship studies suggest that the antimicrobial effect of chalcones is mainly attributable to the presence of hydroxyl phenolic groups (and their high affinity for membrane proteins), especially in the 2′ position of the A ring [23,24]. Furthermore, alkyl group substitution of the A ring, especially *O*-alkyl, is thought to increase lipophilicity, and consequently, enhance antimicrobial activity [25,26,27].

To this end, quantitative structure–activity relationship (QSAR) analysis (i.e., a series of multivariate linear regressions between biological activity (pEC_50_, dependent variable) and various descriptors (physicochemical, steric, or other, independent variables)) was performed following [28,29,30], in order to better describe activity and aid in the design and prediction of new compounds [31].

The multivariate equations obtained in the gaseous and condensed phase (Equations (1) and (2), respectively) showed that the inhibitory activity of 2′-hydroxy-chalcone derivatives on *P. infestans* depends on the molecular area as well as on the atomic charge of carbon C_3′_. Indeed, the atomic charge of chalcone derivatives has previously been discussed for antifungal activity against *B. cinerea* and *M. fructicola* [28,32]. Moreover, the atomic charge of 2-allylphenol derivatives has been related to inhibition activity against other *Phytopthora* strains [32]. Furthermore, while the molecular surface (MS) has been used to explain lipophilicity [7], this descriptor has not been reported as an important feature of *P. infestans* inhibition activity.

pEC_50_ = 5.49 − 0.005MS + 0.464C_3′_ N = 12; r = 0.925; r^2^ = 0.856; SD = 0.032; F = 26.8; q^2^ = 0.832 (1)

pEC_50_ = 5.51 − 0.006MS + 0.472C_3′_ N = 12; r = 0.934; r^2^ = 0.871; SD = 0.029; F = 30.5; q^2^ = 0.852 (2)

The QSAR model obtained in the condensed phase was more robust than that of the gaseous phase (Equation (2) greater values of r, r^2^, F, and q^2^ than Equation (1)). Equation (2) suggests that the growth inhibition activity of 2′-hydroxy-chalcone derivatives against *P. infestans* decreases when molecular surface increases (negative slope MS). However, the most important descriptor is the atomic charge on C_3′_ (~79-folds more than MS). Thus, the atomic charge on C_3′_ very likely provides more active compounds against *P. infestans*.

The most active compounds of the series are **7a**, **8a**, and **9a**; while these compounds have a similar molecular surface, the atomic charge on C_3′_ is different due to the substitution pattern (see Table S2). Thus, new compounds with substitution on C_3′_ were calculated using the compounds **7a**, **8a**, and **9a** as core (see Tables S3–S5). These new compounds differed in calculated pIC_50_, depending on the stereo-electronic properties. For example, using compounds **7a**, **8a**, and **9a** as the core caused the electronegative substituent –F, –OH, and NH_2_ bonded to C_3′_ to increase in positive atomic charge, allowing for increased inhibition growth activity against *P. infestans* (see Tables S3–S5). Interestingly, the derivatives calculated from **7a** showed less activity than derivatives from cores **8a** and **9a**. This phenomenon is likely related to substituent proximity in the **7a** derivatives, which causes stereo-electronic repulsions (see Tables S3–S5). Thus, the most active compound against *P. infestans* (**8a**, see Table 1), the most promising substituents for the inhibition activity of 2-hydroxychalcones against *P. infestans* bonded to C_3′_ were –F and –OH, which allow for ~1.7-folds.

**Figure 1:**
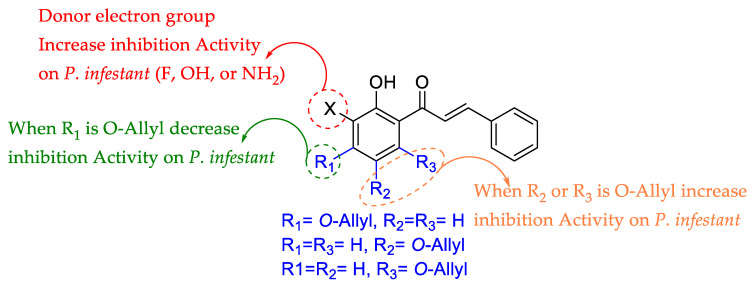
Summary of QSAR analysis.

In sum, QSAR analysis yielded important structural information as well as allowed for calculations of other compounds (see Tables S3–S5) summarized in Figure 1.

## 3. Materials and Methods

### 3.1. Chemicals and Reagents

All chemicals were purchased from Sigma-Aldrich Co. (St. Louis, MO, USA), GIBCO BRL Life Technologies (Grand Island, NY, USA) and Santa Cruz Biotechnology (Santa Cruz, CA, USA). Spectra were recorded in CDCl_3_ solutions and were referenced to the residual peaks of CHCl_3_, δ = 7.26 ppm, and δ = 77.0 ppm for ^1^H and ^13^C, respectively, on a Bruker Avance 400 Digital NMR spectrometer (Bruker, Rheinstetten, Germany), operating at 400.1 MHz for 1 H and 100.6 MHz for ^13^C. Chemical shifts were reported in δ ppm and coupling constants (*J*) were given in Hz. HRMS were measured on Termo Finnigan MAT95XL mass spectrometers. Silica gel (Merck 200–300 mesh) was used for column chromatography.

### 3.2. General Procedure: Synthesis

#### 3.2.1. Alkoxy Acetophenones

A mixture of the respective dihydroxyacetophenone, alkyl bromide, and anhydrous potassium carbonate at a ratio of 1:1.2:1 in 2 mL of acetone and a ratio of 1:2:4 in 2 mL of acetonitrile for monoalkylated and dialkylated chalcones, respectively. The reaction was irradiated in a water bath of an ultrasonic cleaner (Elmasonic S 10 H, Elma Schmidbauer GmbH, Sigen, Germany) with frequency of 37 KHz and a nominal power of 240 W at 45–50 °C for 20–30 min. After the reaction time the extraction, separation and identification protocols was performed according to the reported procedures [33]. This procedure made it possible to obtain known compounds **4a**–**d**, **5a**, **6a**, and **6d** [34,35,36]. NMR data for **4a**–**d**, **5a**, **6a**, and **6d** were consistent with those previously reported. In addition, new compounds such as **5b**–**d** and **6b**–**d** were obtained. The structure of these compounds was established by NMR data.

1-{2-Hydroxy-5-[(2-methylprop-2-en-1-yl)oxy]phenyl}ethanone (**5b**). Dark yellow oil. Yield: 90%. ^1^H NMR (400 MHz, CDCl_3_): δ 11.85 (s, 1H, 2-OH); 7.22 (d, *J* = 3.0 Hz, 1H, H-6); 7.13 (dd, *J* = 9.0 and 3.0 Hz, 1H, H-4); 6.91 (d, *J* = 9.0 Hz, 1H, H-3); 5.10 (s, 1H, H-3α’); 5.01 (s, 1H, H-3β’); 4.42 (s, 2H, H-1′); 2.61 (s, 3H, H-8); 1.84 (s, 3H, H-4′). ^13^C NMR (100 MHz, CDCl_3_): δ 203.9 (C-7); 156.8 (C-2); 150.8 (C-5); 140.9 (C-2′); 125.0 (C-4); 119.2 (C-3); 115.0 (C-6); 113.1 (C-3′); 72.9 (C-1′); 26.7 (C-8); 19.4 (C-4′).

1-[5-(Crotyloxy)-2-hydroxyphenyl]ethanone (**5c**). Brown oil. Yield: 93%. ^1^H NMR (400 MHz, CDCl_3_): δ 11.84 (s, 1H, 2-OH); 7.21 (d, *J* = 3.0 Hz, 1H, H-6); 7.12 (dd, *J* = 9.0 and 3.0 Hz, 1H, H-4); 6.91 (d, *J* = 8.9 Hz, 1H, H-3); 5.87 (m, 1H, H-2′); 5.72 (m, 1H, H-3′); 4.43 (d, *J* = 6.0 Hz, 2H, H-1′); 2.61 (s, 3H, H-8); 1.57 (s, 3H, H-4′). ^13^C NMR (100 MHz, CDCl_3_): δ 205.4 (C-7); 153.7 (C-2); 148.6 (C-5); 129.9 (C-2′); 125.0 (C-3′); 122.4 (C-4); 120.0 (C-1); 119.0 (C-3); 114.0 (C-6); 68.1 (C-1′); 26.7 (C-8); 17.3 (C-4′).

1-[2-Hydroxy-5-(prenyloxy)phenyl]ethanone (**5d**). Dark yellow oil. Yield: 93%. ^1^H NMR (400 MHz, CDCl_3_): *δ* 11.82 (s, 1H, 2-OH); 7.18 (d, *J* = 2.9 Hz, 1H, H-6); 7.10 (dd, *J* = 9.0 and 2.9 Hz, 1H, H-4); 6.88 (d, *J* = 9.0 Hz, 1H, H-3); 5.45 (m, 1H, H-2′); 4.46 (d, *J* = 6.8 Hz, 2H, H-1′); 2.58 (s, 3H, H-8); 1.79 (s, 3H, H-4′); 1.73 (s, 3H, H-5′). ^13^C NMR (100 MHz, CDCl_3_): *δ* 205.5 (C-7); 153.7 (C-2); 148.1 (C-5); 138.4 (C-3′); 122.4 (C-6); 121.4 (C-4); 119.8 (C-2′); 118.9 (C-1); 114.4 (C-3); 65.5 (C-1′); 26.7 (C-8); 25.8 (C-5′); 18.3 (C-4′).

1-{2-Hydroxy-6-[(2-methylprop-2-en-1-yl)oxy]phenyl}ethanone (**6b**). Yellow oil. Yield: 86%. ^1^H NMR (400 MHz, CDCl_3_): δ 13.24 (s, 1H, 2-OH); 7.31 (t, *J* = 8.3 Hz, 1H, H-4); 6.57 (d, *J* = 8.2 Hz, 1H, H-3); 6.38 (d, *J* = 8.3 Hz, 1H, H-5); 5.1 (s, 1H, H-3α’); 5.05 (s, 1H, H-3β’); 4.52 (s, 2H, H-1′); 2.71 (s, 3H, H-8); 1.87 (s, 3H, H-4′). ^13^C NMR (100 MHz, CDCl_3_): δ 202.5 (C-7); 162.0 (C-2); 155.4 (C-6); 139.1 (C-2′); 136.0(C-4); 113.8 (C-1); 111.6 (C-3′); 111.1 (C-5); 101.5 (C-3); 72.4 (C-1′); 33.2 (C-8); 18.8 (C-4′).

1-[6-(Crotyloxy)-2-hydroxyphenyl]ethanone (**6c**). Brown oil. Yield: 86%. ^1^H NMR (400 MHz, CDCl_3_): δ 13.34 (s, 1H, 2-OH); 7.12 (d, *J* = 8.3 Hz, 1H, H-4); 6.57 (d, *J* = 8.2 Hz, 1H, H-3); 6.34 (d, *J* = 8.3 Hz, 1H, H-5); 5.62 (m, 2H, H-2′ and H-3′); 4.38 (d, J = 5.6 Hz, 2H, H-1′); 2.72 (s, 3H, H-8); 1.72 (s, 3H, H-4′). ^13^C NMR (100 MHz, CDCl_3_): δ 202.5 (C-7); 162.0 (C-2); 159.8 (C-6); 136.0 (C-4); 127.8 (C-2′); 125.0 (C-3′); 112,0 (C-1); 111.1 (C-5); 106.8 (C-3); 64.0 (C-1′); 33.2 (C-8); 13.3 (C-4′).

#### 3.2.2. Oxyalkyl Chalcones

To a solution of benzaldehyde (1.5 mmol) and the corresponding oxyalkyl acetophenone (1.5 mmol) in ethanol (3 mL) taken in a flask (25 mL), a catalytic quantity of sodium hydroxide (0.75 mmol) was added and the reaction mixture was irradiated in the water bath of an ultrasonic cleaner with a frequency of 37 KHz and a nominal power of 240 W at 30–35 °C for 3 h, after which 10% HCl solution was added until pH ~ 7 to end the reaction, and the mixture was extracted with EtOAc (3 × 30 mL). The organic layer was dried with Na_2_CO_3_, filtered, and separated with column chromatography on silica gel.

This procedure made it possible to obtain four known chalcones, **7a**, **7b**, **7c**, and **7d**, with high percentages of yield 9%, 85.3%, 86.0%, and 81.1%, respectively. NMR data for **7a**–**7d** were consistent with those previously reported [37]. The structure of the new compounds obtained was established by NMR and mass data, as detailed below:

(2E)-1-[5-(Allyloxy)-2-hydroxyphenyl]-3-phenylprop-2-en-1-one (**8a**). Solid orange. Yield: 91%. m.p.: 59 °C. ^1^H NMR (400 MHz, CDCl_3_): δ 12.37 (s, 1H, 2′-OH); 7.92 (d, *J* = 15.5 Hz, 1H, H-7); 7.67 (m, 2H, H-2 and H-6); 7.59 (d, *J* = 15.5 Hz, 1H, H-8); 7.44 (m, 3H, H-3, H-4 and H-5); 7.41 (s, 1H, H-6′); 7.17 (d, *J* = 9.0 Hz, H-4′); 6.98 (d, *J* = 9.0 Hz, 1H, H-3′); 6.08 (m, 1H, H-2″); 5.45 (d, *J* = 17.2 Hz, 1H, H-3_α_″); 5,32 (d, *J* = 10.6 Hz, 1H, H-3_β_″); 4.56 (d, *J* = 5.3 Hz, 2H, H-1″). ^13^C NMR (100 MHz, CDCl_3_): *δ* 193.4 (C-9); 158.1 (C-2′); 150.7 (C-5′); 145.6 (C-7); 134.6 (C-1); 133.2 (C-2″); 131.0 (C-4); 129.1 (C-3 and C-5); 128.7 (C-2 and C-6); 124.7 (C-4′); 120. 1 (C-8); 119.7 (C-1′); 119.3 (C-3′); 118.0 (C-3″); 114.5 (C-6′); 70.0 (C-1″). HREIMS: M^+^H ion m/z 280.3179 (C_18_H_16_O_3_: 280.3178).

(2E)-1-{2-Hydroxy-5-[(2-methylprop-2-en-1-yl)oxy]phenyl}-3-phenylprop-2-en-1-one (**8b**). Dark yellow oil. Yield: 90%. ^1^H NMR (400 MHz, CDCl_3_): δ 12.36 (s, 1H, 2′-OH); 7.92 (d, *J* = 15.5 Hz, 1H, H-7); 7.67 (m, 2H, H-2 and H-6); 7.59 (d, *J* = 15.5 Hz, 1H, H-8); 7.45 (m, 3H, H-3, H-4, and H-5); 7.42 (s, 1H, H-6′); 7.16 (d, *J* = 9.0 Hz, 1H, H-4′); 6.97 (d, *J* = 9.0 Hz, 1H, H-3′); 5.14 (s, 1H, H-3_β_″); 5.04 (s, 1H, H-3_α_″); 4.46 (s, 2H, H-1″); 1.86 (s, 3H, H-4″). ^13^C NMR (100 MHz, CDCl_3_): δ 193.4 (C-9); 158.0 (C-2′); 150.8 (C-5′); 145.5 (C-7); 141.0 (C-2″); 134.6 (C-1); 130.9 (C-4); 129.1 (C-3 and C-5); 128.6 (C-2 and C-6); 124.7 (C-4′); 120.2 (C-8); 119.7 (C-1′); 119.2 (C-3′); 114.3 (C-6′); 113.1 (C-3″); 73.0 (C-1″); 19.4 (C-4″). HRMS: M^+^H ion m/z 294.3447 (C_19_H_18_ O_3_: 294.3444).

(2E)-1-[5-(Crotyloxy)-2-hydroxyphenyl]-3-phenylprop-2-en-1-one (**8c**). Solid red. Yield: 89%. m.p.: 104 °C. ^1^H RMN (400 MHz, CDCl_3_): δ 12.35 (s, 1H, 2′-OH); 7.92 (d, *J* = 15.5 Hz, 1H, H-7); 7.66 (m, 2H, H-2 and H-6); 7.60 (d, *J* = 15.5 Hz, 1H, H-8); 7.45 (m, 3H, H-3, H-4, and H-5); 7.39 (s, 1H, H-6′); 7.16 (d, *J* = 9.0 Hz, 1H, H-4′); 6.97 (d, *J* = 9.0 Hz, 1H, H-3′); 5.91 (m, 1H, H-2″); 5.84 (m, 1H, H-3″); 4.46 (d, *J* = 6.0 Hz, 2H, H-1″); 1.56 (s, 3H, H-4″). ^13^C NMR (100 MHz, CDCl_3_): *δ* 192.0 (C-9); 156.3 (C-2′); 153.3 (C-5′); 145.3 (C-7); 138.9 (C-1); 130.9 (C-4); 129.0 (C-2″); 128.8 (C-3 and C-5); 128.7 (C-2 and C-6); 126.0 (C-3″); 125.7 (C-4′); 120.7 (C-8); 119.4 (C-3′); 108.6 (C-6′); 69.3 (C-1″); 17.9 (C-4″). HRMS: M^+^H ion m/z 294.3448 (C_19_H_18_O_3_: 294.3445).

(2E)-1-[2-Hydroxy-5-(prenyloxy)phenyl]-3-phenylprop-2-en-1-one (**8d**). Solid pale brown. Yield: 85%. m.p.: 63.5 °C. ^1^H NMR (400 MHz, CDCl_3_): *δ* 12.35 (s, 1H, 2′-OH); 7.92 (d, *J* = 15.5 Hz, 1H, H-7); 7.66 (m, 2H, H-2, and H-6); 7.60 (d, *J* = 15.5 Hz, 1H, H-8); 7.45 (m, 3H, H-3, H-4, and H-5); 7.39 (s, 1H, H-6′); 7.16 (m, 1H, H-4′); 6.97 (d, *J* = 9.0 Hz, 1H, H-3′); 5.51 (m, 1H, H-2″); 4.53 (d, *J* = 6.7 Hz, 2H, H-1″); 1.82 (s, 3H, H-4″); 1.77 (s, 3H, H-5″). ^13^C NMR (100 MHz, CDCl_3_): δ 193,4 (C-9); 157,9 (C-2′); 150,9 (C-5′); 145,5 (C-7); 138,4 (C-3″); 134.6 (C-1); 130.9 (C-4); 130.6 (C-4); 129.1 (C-3 and C-5); 128.6 (C-2 and C-6); 126.6 (C-4′); 120.2 (C-8); 119.7 (C-2″); 119.5 (C-3′); 114.4 (C-6′); 65.8 (C-1″); 25.8 (C-5″); 18.3 (C-4″). HRMS: M^+^H ion m/z 308.3715 (C_20_H_20_O_3_: 308.3710).

(2E)-1-[2-(Allyloxy)-6-hydroxyphenyl]-3-phenylprop-2-en-1-one (**9a**). Solid dark yellow. Yield: 90%. m.p.: 78.5 °C. ^1^H NMR (400 MHz, CDCl_3_): δ 13.39 (s, 1H, 2′-OH); 7.95 (d, *J* = 15.6 Hz, 1H, H-7); 7.81 (d, *J* = 15.6 Hz, 1H, H-8); 7.61(m, 2H, H-2, and H-6); 7.39 (m, 3H, H-3, H-4 and H-5); 7.23 (m, 1H, H-4′); 6.62 (d, *J* = 8.5 Hz, 1H, H-5′); 6.38 (d, *J* = 8.5 Hz, 1H, H-3′); 5.99 (m, 1H, H-2″); 5.47 (d, *J* =17.2 Hz, 1H, H-3_α_″); 5.32 (d, *J* = 10.6 Hz, 1H, H-3_β_″); 4.63 (d, *J* = 5.4 Hz, 2H, H-1″). ^13^C RMN (100 MHz, CDCl_3_): δ 194.8 (C-9); 162.3 (C-2′); 158.4 (C-6′); 142.6 (C-7); 136.6 (C-1); 135.8 (C-4′); 132.5 (C-2″); 130.2 (C-4); 128.8 (C-3 and C-5); 128.5 (C-2 and C-6); 127.9 (C-8); 118.6 (C-3″); 115.6 (C-3′); 110.8 (C-1′); 102.1 (C-5′); 69.7 (C-1″). HRMS: M^+^H ion m/z 280.3173 (C_18_H_16_O_3_: 280.3178).

(2E)-1-{2-Hydroxy-6-[(2-methylprop-2-en-1-yl)oxy]phenyl}-3-phenylprop-2-en-1-one (**9b**). Solid dark green. Yield: 83%. m.p.: 68–69 °C. ^1^H NMR (400 MHz. CDCl_3_): 13.02 (s, 1H, 2′-OH); 7.92 (d, *J* = 15.6 Hz, 1H, H-7); 7.81 (d, *J* = 15.6 Hz, 1H, H-8); 7. 59 (m, 2H, H-2 and H-6); 7.38 (m, 3H, H-3, H-4 and H-5); 7.33 (m, 1H, H-4′); 6.62 (d, *J* = 8.5 Hz, 1H, H-5′); 6.42 (d, *J* = 8.5 Hz, 1H, H-3′); 5.17 (s, 1H, H-3_β_″); 5.04 (s, 1H, H-3α″); 4.54 (s, 2H, H-1″); 1.83 (s, 3H, H-4″). ^13^C NMR (100 MHz, CDCl_3_): *δ* 194.5 (C-9); 164.7 (C-2′); 160.1 (C-6′); 142.8 (C-7); 140.1 (C-2″); 135.9 (C-4′); 135.2 (C-1); 130.3 (C-4); 128.8 (C-3 and C-5); 128.6 (C-2 and C-6); 127.8 (C-8); 114.1 (C-3″); 111.0 (C-3′); 102.5 (C-5′); 73.0 (C-1″); 19.7 (C-4″). HRMS: M^+^H ion m/z 294.3441 (C_19_H_18_ O_3_: 294.3444).

(2E)-1-[6-(Crotyloxy)-2-hydroxyphenyl]-3-phenylprop-2-en-1-one (**9c**). Red oil. Yield: 86%. ^1^H NMR (400 MHz, CDCl_3_): δ 13.25 (s, 1H, 2′-OH); 8.00 (d, *J* = 15.6 Hz, 1H, H-7); 7.79 (d, *J* = 15.6 Hz, 1H, H-8); 7.61 (m, 2H, H-2 and H-6); 7.39 (m, 3H, H-3, H-4 and H-5); 7.33 (m, 1H, H-4′); 6.61 (d, *J* = 8.5 Hz, 1H, H-5′); 6.41 (d, *J* = 8.5 Hz, 1H, H-3′); 5.94 (m, 1H, H-2″); 5.82 (m, 1H, H-3″); 4.56 (d, *J* = 6.0 Hz, 2H, H-1″); 1.77 (s, 3H, H-4″). ^13^C RMN (100 MHz, CDCl_3_): δ 194.6 (C-9); 165.0 (C-2′); 160.2 (C-6′); 142.6 (C-7); 135.9 (C-4′); 135.4 (C-1); 131.7 (C-4); 130.2 (C-2″); 128.9 (C-3 and C-5); 128.5 (C-2 and C-6); 128.1 (C-8); 125.3 (C-3″); 112.0 (C-3′); 111.0 (C-1′); 102.5 (C-5′); 69.6 (C-1″); 17.9 (C-4″). HRMS: M^+^H ion m/z 294.3447 (C_19_H_18_O_3_: 294.3445).

(2E)-1-[2-Hydroxy-6-(prenyloxy)phenyl]-3-phenylprop-2-en-1-one (**9d**). Solid dark yellow. Yield: 80%. m.p.: 54.3 °C. ^1^H NMR (400 MHz, CDCl_3_): δ 13.42 (s, 1H, 2′-OH); 8.06 (d, *J* = 15.6 Hz, 1H, H-7); 7.78 (d, *J* = 15.6 Hz, 1H, H-8); 7.60 (m, 2H, H-2 and H-6); 7.38 (m, 3H, H-3, H-4 and H-5); 7.34 (m, 1H, H-4′); 6.61 (d, *J* = 8.5 Hz, 1H, H-5′); 6.44 (d, *J* = 8.5 Hz, 1H, H-3′); 5.62 (m, 1H, H-2″); 4.60 (d, *J* = 6.7 Hz, 2H, H-1″); 1.84 (s, 3H, H-4″); 1.77 (s, 3H, H-5″). ^13^C NMR (100 MHz, CDCl_3_): δ 194.7 (C-9); 165.3 (C-2′); 160.6 (C-6′); 142.6 (C-7); 139.7 (C-4′); 136.0 (C-3″); 135.5 (C-1); 130.1 (C-4); 128.8 (C-3 and C-5); 128.4 (C-2 and C-6); 128.0 (C-8); 118.8 (C-2″); 111.9 (C-3′); 110.8 (C-1′); 102.4 (C-5′); 65.6 (C-1″); 25.8 (C-5″); 18.2 (C-4″). HRMS: M^+^H ion m/z 308.3712 (C_20_H_20_O_3_: 308.3710).

### 3.3. Anti-Oomycete Activity

All oxyalkyl chalcones **7a**–**d**, **8a**–**d**, and **9a**–**d** were subjected to antifungal assays against *Phytophthora infestans*, obtained from the collection maintained in our Laboratory of Biochemistry and Environmental Microbiology in Chillan, Chile.

Mycelial discs (4 mm in diameter) of test fungi grown on rye B agar [38] were cut from the margins of the colony and placed on the same medium containing different concentrations of the test oxyalkyl chalcones (12.5–400 µg/mL). After incubation at 20 °C for seven days, radial mycelial growth was measured and was compared with the controls [39]. Control plates were treated with difenoconazole and metalaxil. Activity was expressed as EC_50_ (the concentration inhibiting growth by 50%), MIC (the minimum concentration inhibition of mycelial growth), and MOC was defined as the lowest concentration of the chemicals that prevented visible growth or germination of mycelium. The experiments were repeated three times with three replicates.

### 3.4. Membrane Damage

*P. infestans* strains were cultured by shaking at 20 °C and then washed twice and diluted to approximately (3 × 10^4^ zoospores/mL with cold 3-(N-morpholino)propanesulfonic acid (MOPS) buffer, pH 6.0. Cells were aliquoted to tubes, and **7a**–**d**, **8a**–**d**, and **9a**–**d** was added at a final concentration of 100 µg/mL. SDS (2%) was used as the reference compound, which produced 100% cellular oomycete leakage. *P. infestans* was incubated at 20 °C, and samples were taken at time intervals (6, 12, 24, and 48 h) and spun at 3500 rpm for 7 min in microcentrifugetubes. The supernatants were collected for absorbance analysis at 260 nm in a Beckman DU-600 spectrophotometer [37]. Results are the means of values from at least two independent assays.

### 3.5. Computational Details

All compounds (**7a**–**d**, **8a**–**d**, and **9a**–**d**) were optimized using DFT-B3LYP-6-31G (d,p) level of theory calculations, and the optimized structures were verified by frequency calculations (obtaining no imaginary frequencies) in the gas phase and using the integral equation formalism (IEFPCM) (water) model as the solvent phase according to a previous report [27]. The descriptors obtained from quantum mechanical calculations such as the dipolar moment (DM), atomic charge from the electrostatic potential (C_1_, C_2_, C_3_, C_4_, C_5_, C_6_, C_1′_, C_2′_, C_3′_, C_4′_, C_5′_, C_6′_, Cα, Cβ, CO), highest occupied molecular orbital (HOMO), and lowest unoccupied molecular orbital (LUMO) were obtained directly from the output file, while the chemical potential (µ), hardness (η), softness (S), and electrophilic global index (ω) values were calculated using the following equations.

(3) $$\mu =\frac{\left(E_{LUMO}+E_{HOMO}\right)}{2}$$

(4) $$\eta =\;\frac{\left(E_{LUMO}-E_{HOMO}\right)}{2}$$

(5) $$S=\;\frac{2}{2\eta }$$

(6) $$\omega =\frac{\mu^{2}}{2\eta }$$

In addition, steric and topological descriptors such as molecular weight (MW), molecular surface (MS), molecular volume (MV), hydrogen bonding acceptor (HA), hydrogen bonding donor (HD), rotatable bonds (RT), and topological diameter (TD), lipophilicity index (CLogP), molar refractivity (MR) were obtained using molecular mechanics (MM) optimization carried out with the ChemDraw software.

### 3.6. Quantitative Structure–Activity Relationship (QSAR)

The structure–activity relationship study was carried out using multiple linear regressions as described in our previous report with small changes [30,40]. We developed several regression models using pEC_50_ (−log_10_(EC_50_)) in M units as the dependent variable and all descriptors above-mentioned in the gas phase and in the solvent phase as independent variables (DM, C_1_, C_2_, C_3_, C_4_, C_5_, C_6_, C_1′_, C_2′_, C_3′_, C_4′_, C_5′_, C_6′_, Cα, Cβ, CO, HOMO, LUMO, ΔLH, µ, η, S, ω, MW, MS, MV, HA, HD, RT, TD, CLogP, MR). In addition, to avoid random correlations between pEC_50_ and any descriptor, cross-validation was carried out using the Golbraikh method as described in Equation (7) [41]:

(7) $$q^{2}=1-\frac{\sum {\left(y_{obs}-y_{calc}\right)}^{2}}{\sum {\left(y_{obs}-y_{ave}\right)}^{2}}$$

where *y_obs_* is the experimental pEC_50_; *y_calc_* is the pEC_50_ calculated by the QSAR model; and *y_ave_* is the average pEC_50_ of all of the compounds used in the QSAR model. An acceptable value of *q*^2^ was equal to or higher than 0.5.

### 3.7. Statistical

In vitro assays were performed in triplicate and the results expressed as mean values ± SD. The results were analyzed using the standard method [39].

## 4. Conclusions

The results of this research show that the presence of a short alkyl chain improves biological activity. Data so far suggest that oxyalkylated chalcones may represent a new frontier and a challenge for the development of new anti-oomycete compounds against *Phytophthora* spp. in the near future. Indeed, our results show that research into 2′-hydroxy-chalcone derivate compounds can generate new applications with important impacts for productive sectors, specifically Solanaceae crops (tomatoes, potatoes, and chili).

## Figures and Tables

**Table 1:** Inhibition of mycelial growth by 2′-hydroxy-chalcone derivatives against *P. infestans.*

Compound	^a^ EC_50_	^b^ MIC	^c^ MOC
**7a**	44.0 ± 0.3	25	50
**7b**	67.0 ± 0.4	50	75
**7c**	67.4 ± 0.2	50	75
**7d**	75.9 ± 0.3	50	75
**8a**	32.9 ± 0.6	12.5	25
**8b**	53.5 ± 1.0	25	50
**8c**	53.3 ± 0.2	25	50
**8d**	67.0 ± 2.3	50	75
**9a**	44.0 ± 1.7	25	50
**9b**	57.8 ± 0.2	50	75
**9c**	55.6 ± 0.3	50	75
**9d**	65.9 ± 0.2	50	75
**Difenoconazole**	113.8 ± 0.4	100	125
**Metalaxyl**	28.6 ± 0.7	12.5	12.5

^a^ The concentration inhibiting growth by 50% (µg/mL) and ± is the standard deviation from three independent experiments. ^b^ The minimum inhibitory concentration (µg/mL). ^c^ The minimum oomycetidal concentration (µg/mL).

**Table 2:** Percentage of membrane lysis of the synthetic compounds **7a**–**d**, **8a**–**d,** and **9a**–**d**.

Compounds	*%* Membrane Lysis ^a^
**7a**	50.1 ± 0.3
**7b**	31.1 ± 0.6
**7c**	29.0 ± 0.5
**7d**	27.3 ± 0.3
**8a**	100 ± 0.0
**8b**	47.0 ± 0.1
**8c**	45.0 ± 0.3
**8d**	30.0 ± 0.2
**9a**	55.0 ± 0.2
**9b**	42.7 ± 0.4
**9c**	41.2 ± 0.3
**9d**	33.0 ± 0.1
**SDS**	100 ± 0.0

^a^ The assay was performed in triplicate.

## Supplementary Materials

The following are available online at https://www.mdpi.com/2079-6382/9/9/576/s1, SpectraS1: ^1^ H and ^13^ C NMR of compounds 5b–5d and 6b–6c. SpectraS2: ^1^H, ^13^C NMR, and MS of compounds 8a–8d and 9a–9d, Table S1: Result of quantitative structure–activity relationship in gas phase, Table S2: Result of quantitative structure activity relationship in condensed phase, Table S3: Proposal compounds based in QSAR analysis based in 7a core, Table S4: Proposal compounds based in QSAR analysis based in 8a core, Table S5: Proposal compounds based in QSAR analysis based in 9a core.
