# Supplementary material for: Sonochemical Synthesis of 2’-Hydroxy-Chalcone Derivatives with Potential Anti-Oomycete Activity
Source: Antibiotics (Basel). 2020 Sep 4;9(9):576. doi: 10.3390/antibiotics9090576 (PMC7560025; doi:10.3390/antibiotics9090576)
Supplement: Supplementary file 1 [file antibiotics-09-00576-s001.pdf]

SpectraS1: ^{1}H and ^{13}C NMR of compounds 5b–5d and 6b-6c

^{1}H NMR (400 MHz, $CDCl_3$) spectrum of compound 5b

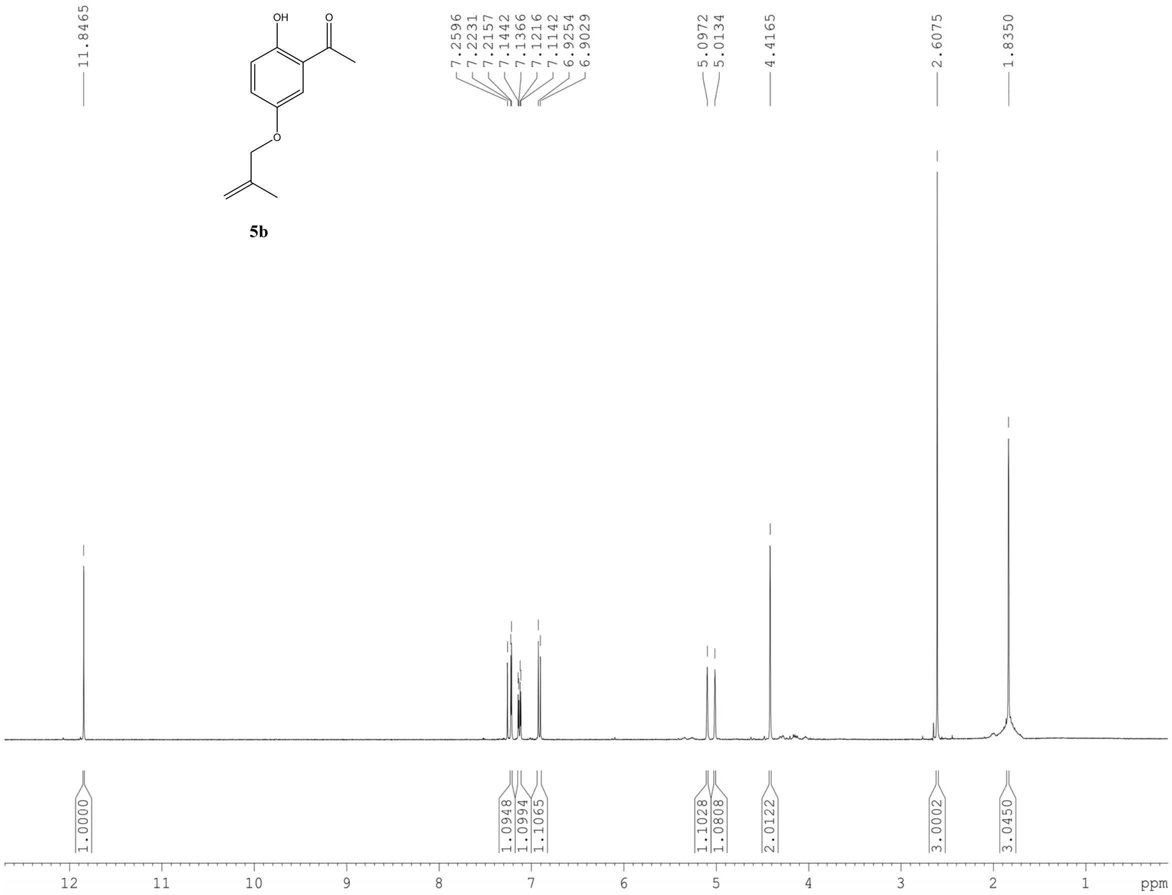

^{13}C NMR (100 MHz, $CDCl_3$) spectrum of compound 5b

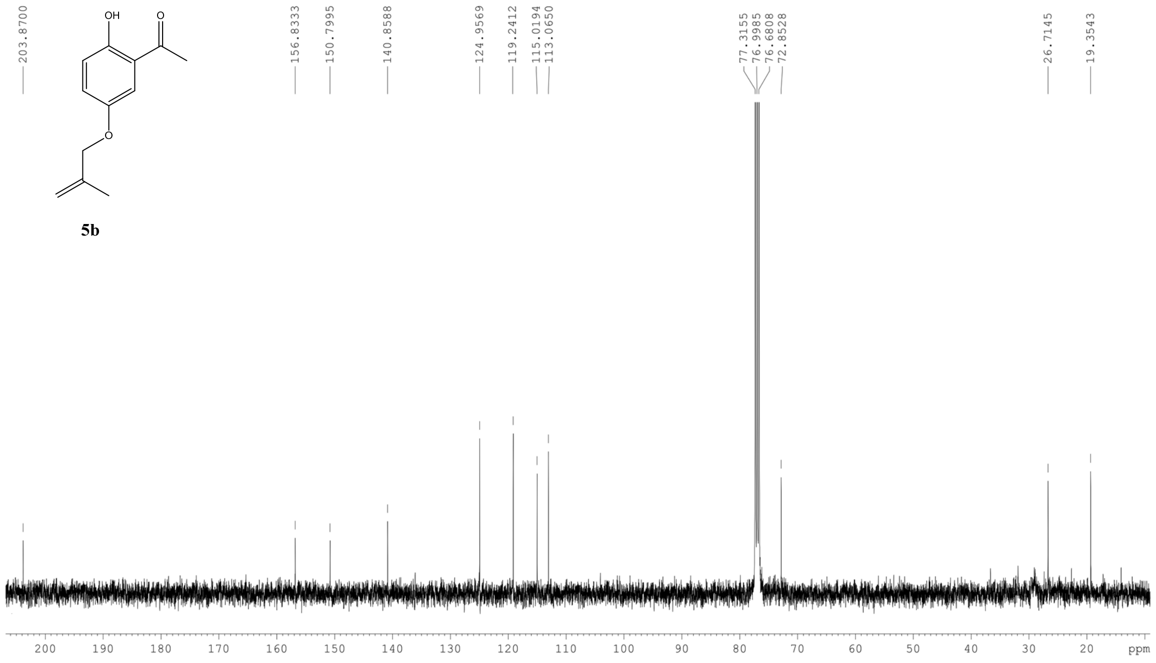

5b

^{1}H NMR (400 MHz, $CDCl_3$) spectrum of compound 5c

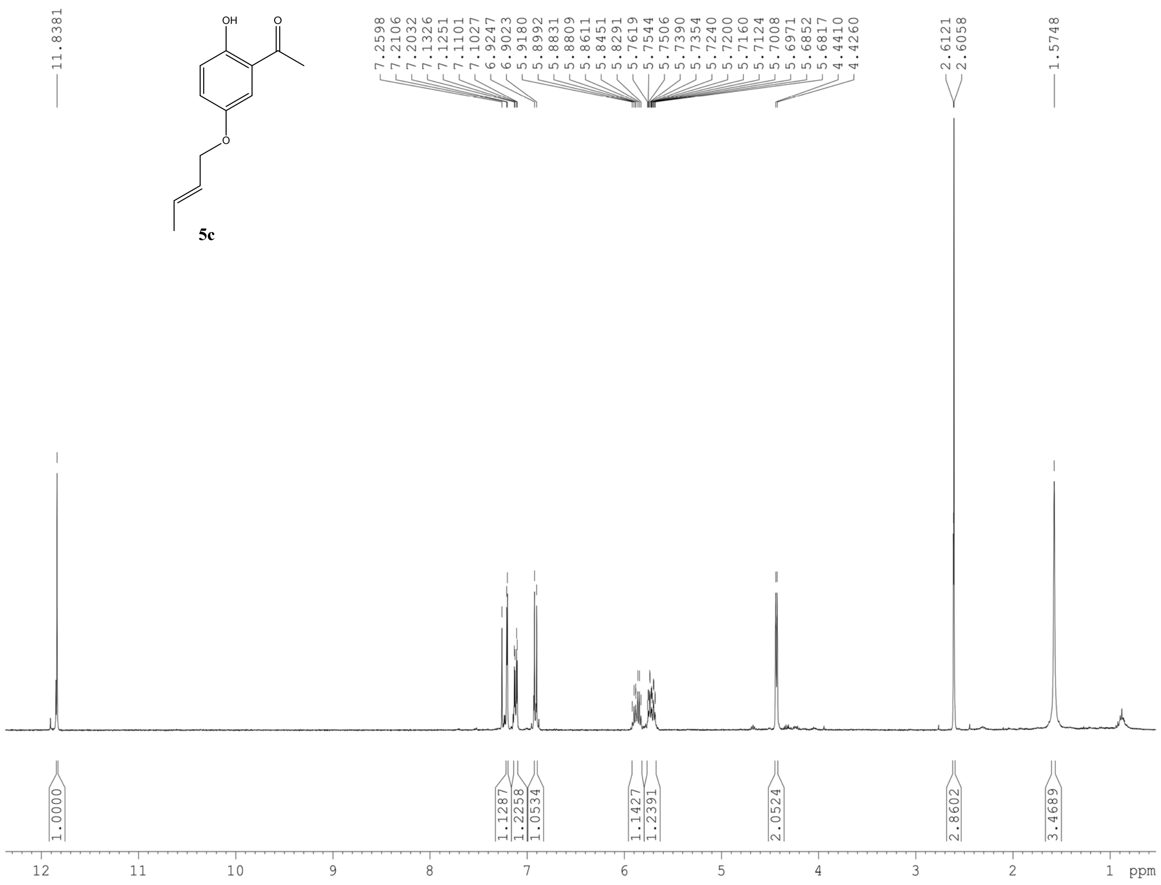

^{13}C NMR (100 MHz, $CDCl_3$) spectrum of compound 5c

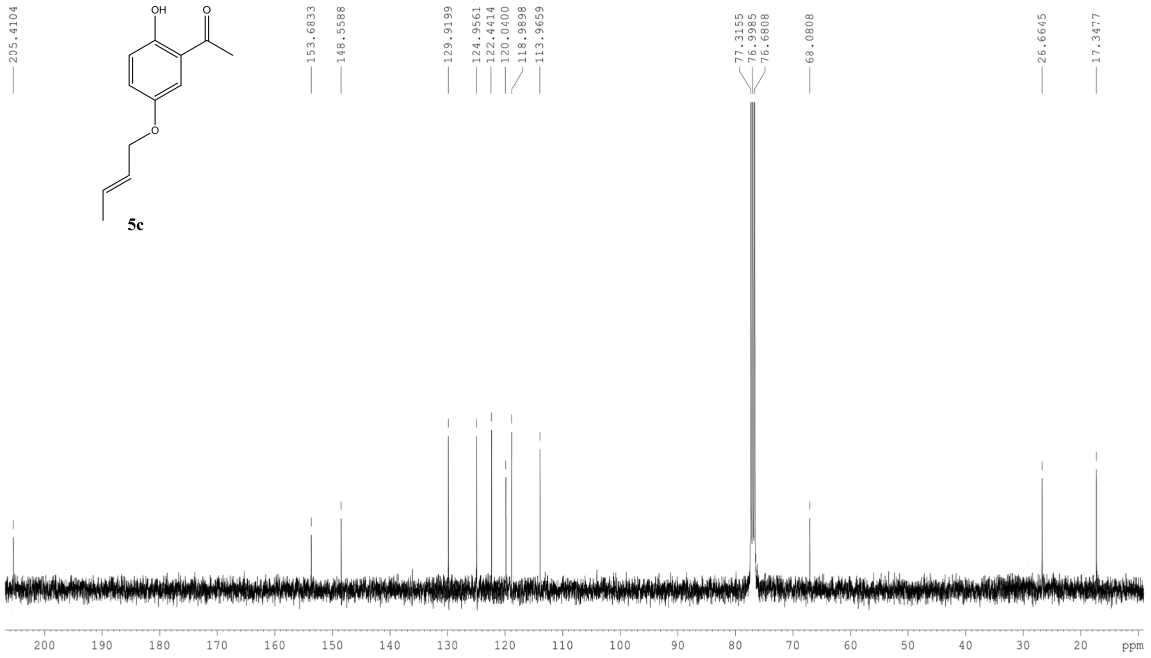

^{1}H NMR (400 MHz, $CDCl_3$) spectrum of compound 5d

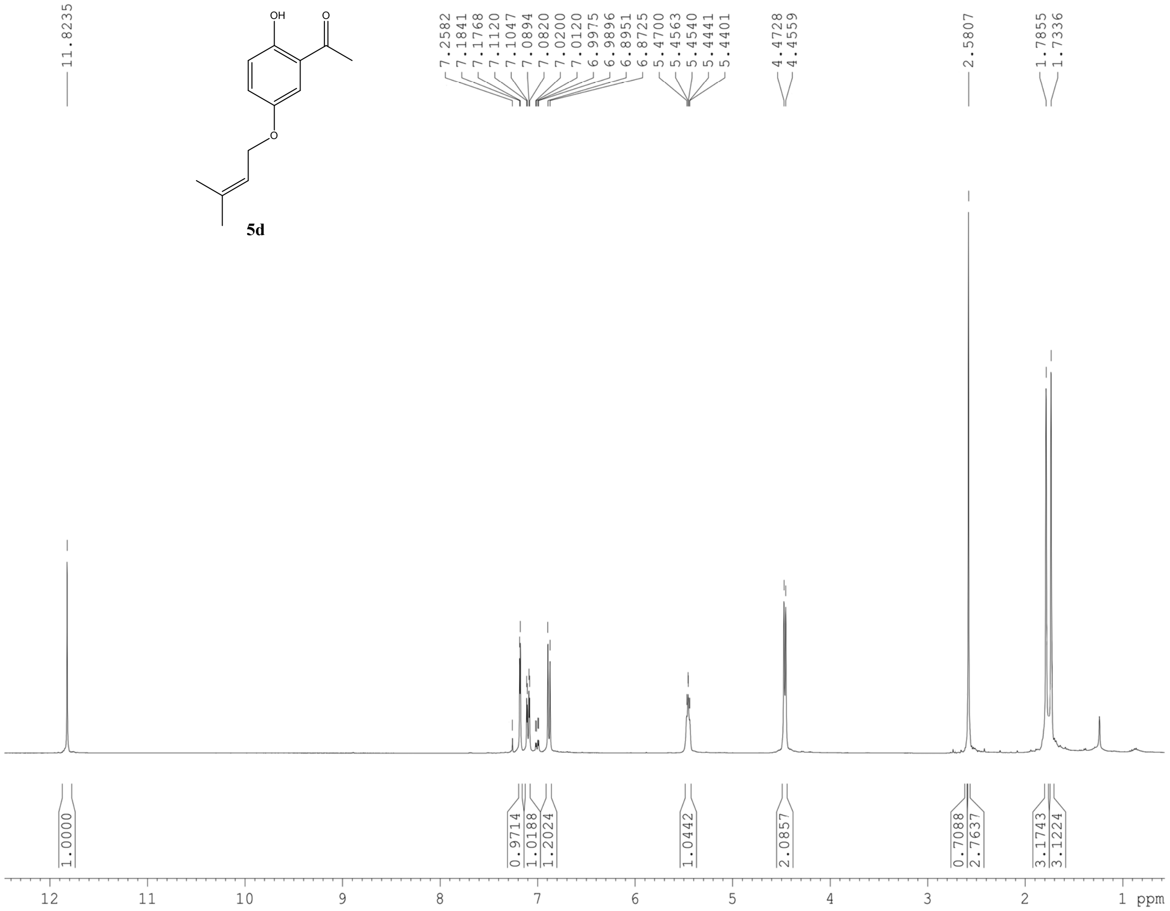

^{13}C NMR (100 MHz, CDCl3) spectrum of compound 5d

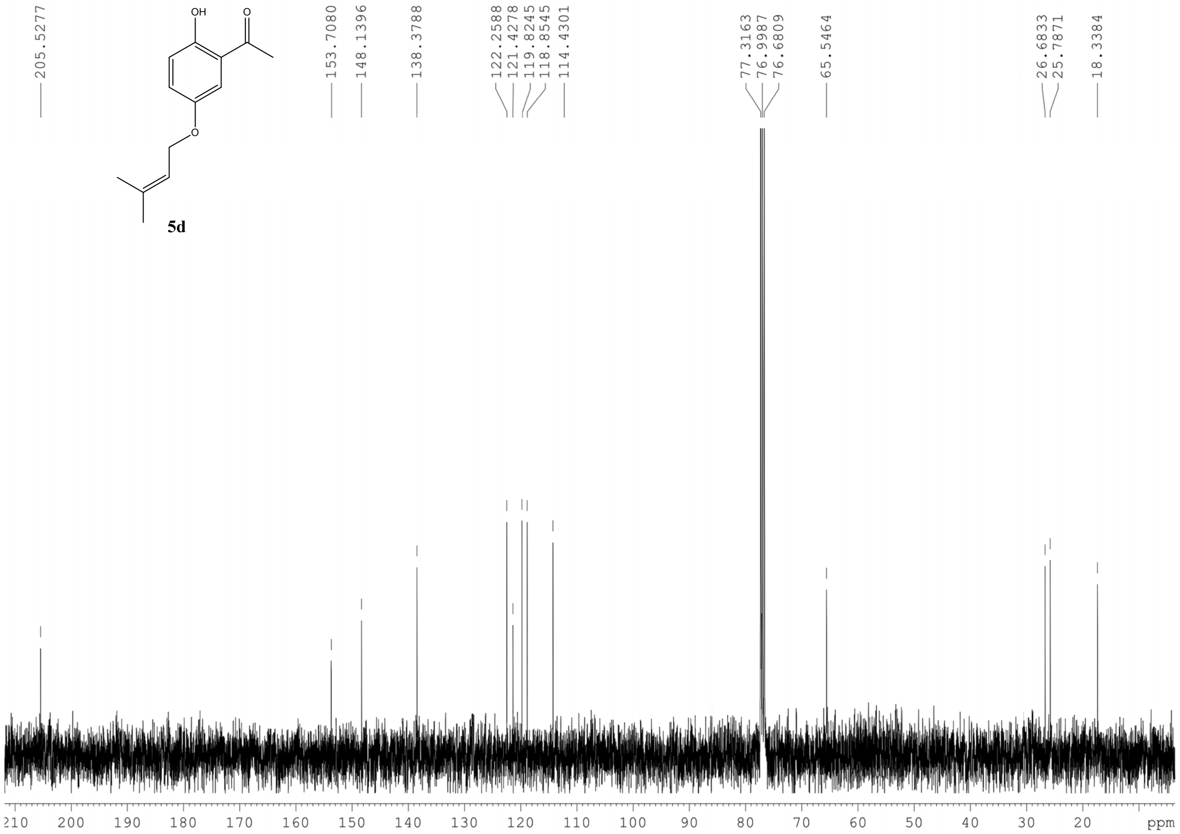

^{1}H NMR (400 MHz, $CDCl_3$) spectrum of compound 6b

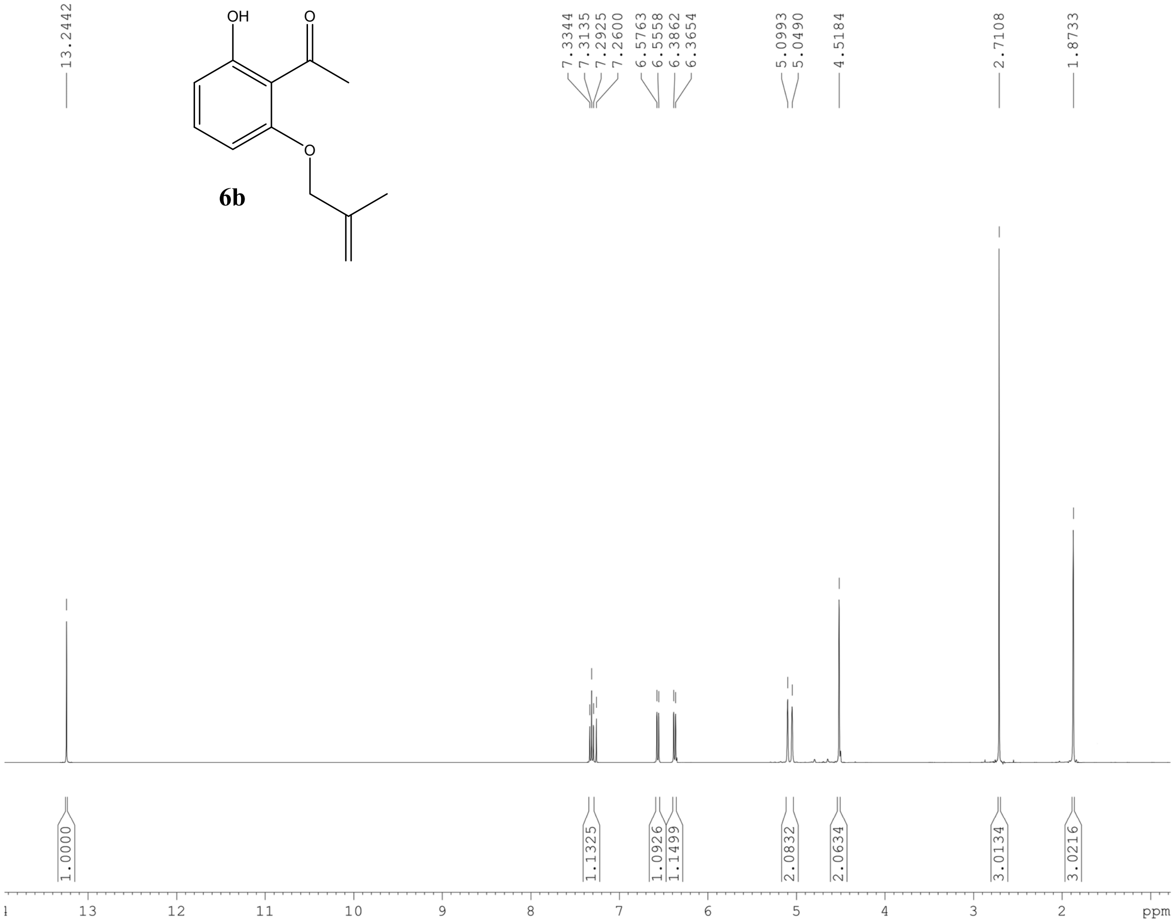

^{13}C NMR (100 MHz, $CDCl_3$) spectrum of compound 6b

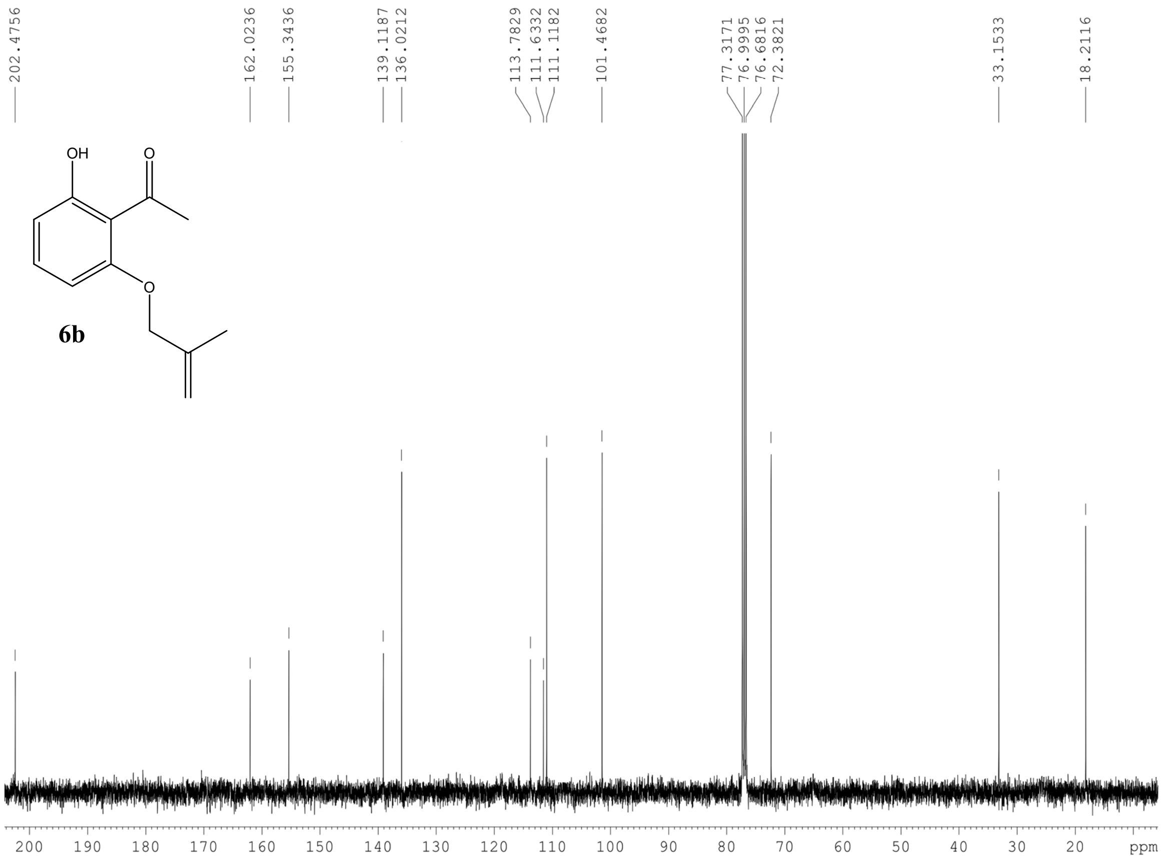

^{1}H NMR (400 MHz, $CDCl_3$) spectrum of compound 6c

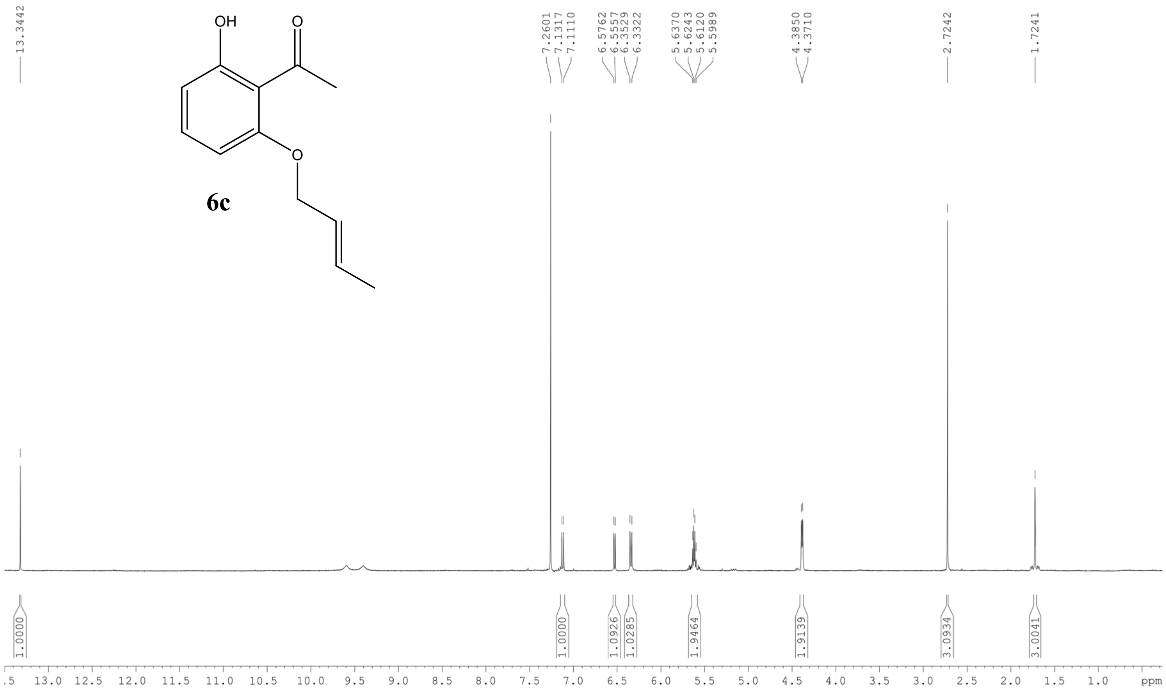

^{13}C NMR (100 MHz, $CDCl_3$) spectrum of compound 6c

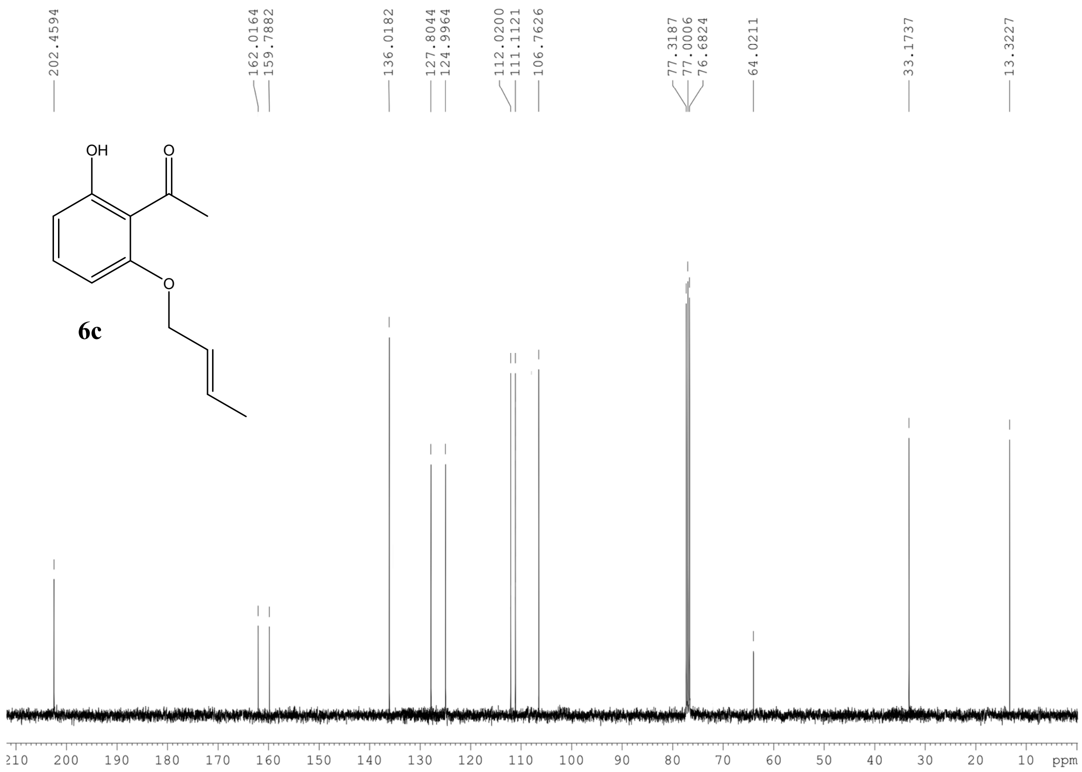

SpectraS2: ^{1}H, ^{13}C NMR, and MS of compounds 8a-8d and 9a-9d

^{1}H NMR (400 MHz, $CDCl_3$) spectrum of compound 8a

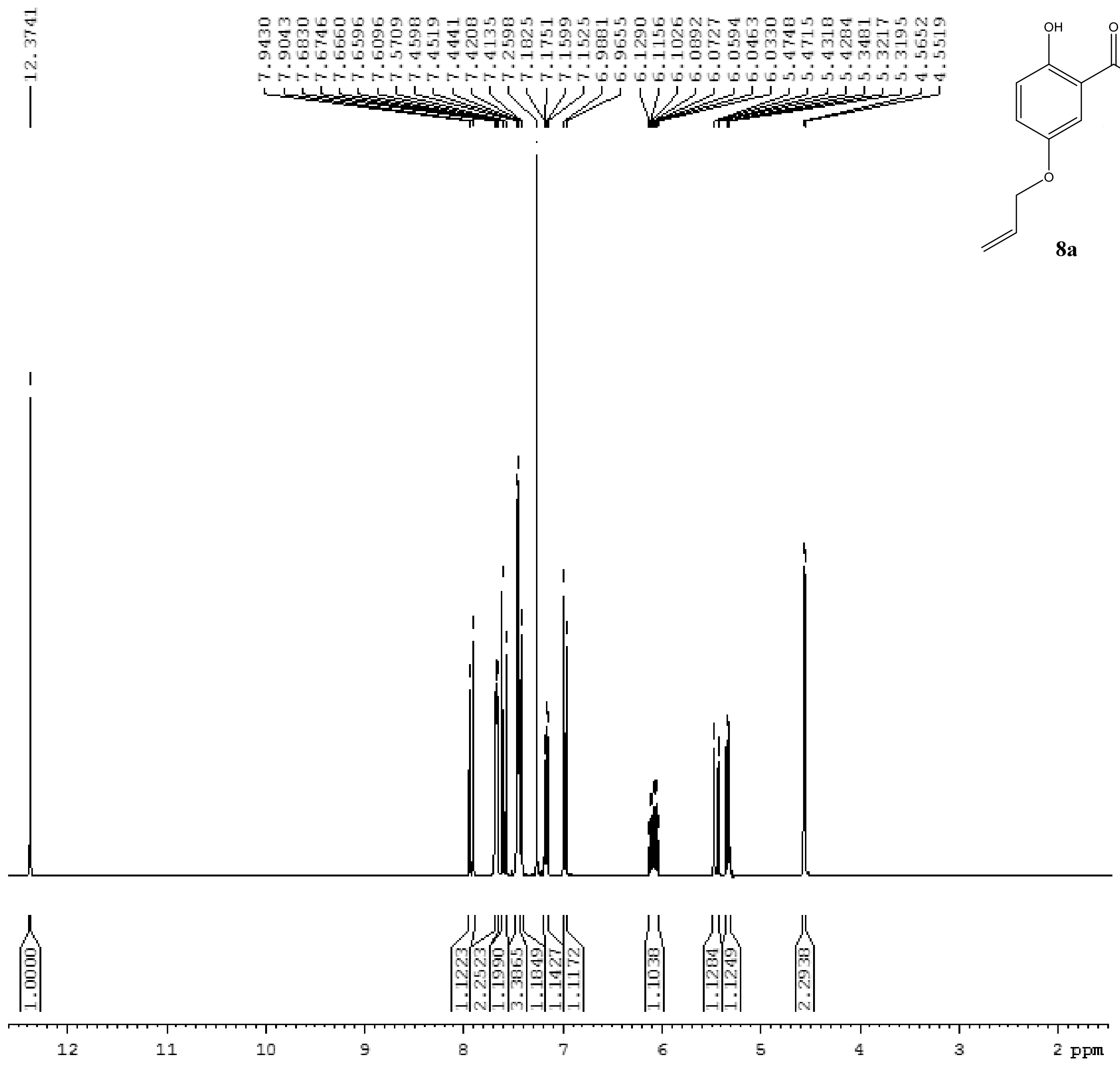

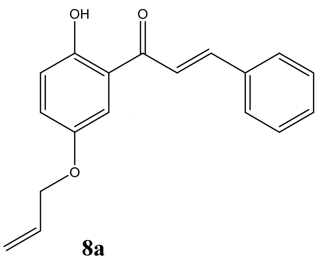

8a

^{13}C NMR (100 MHz, $CDCl_3$) spectrum of compound 8a

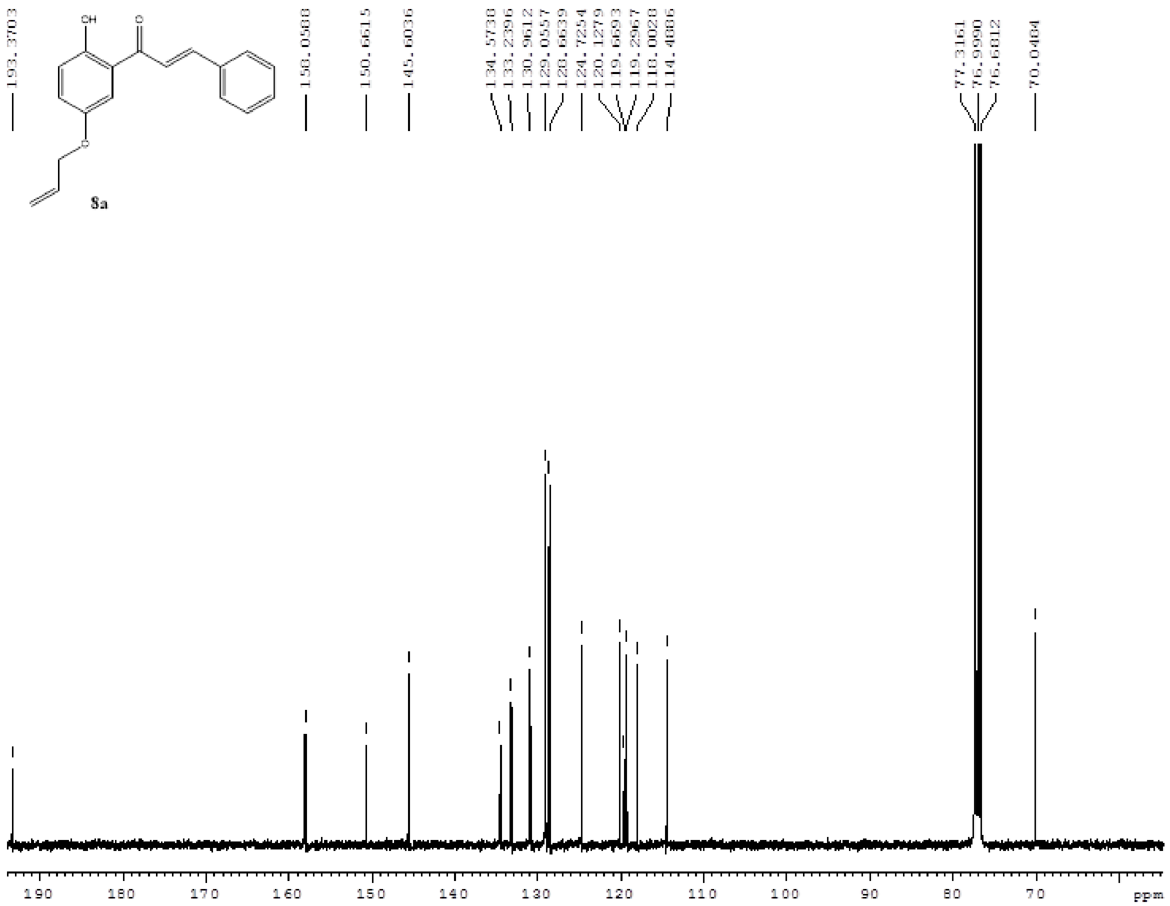

Mass of compound 8a

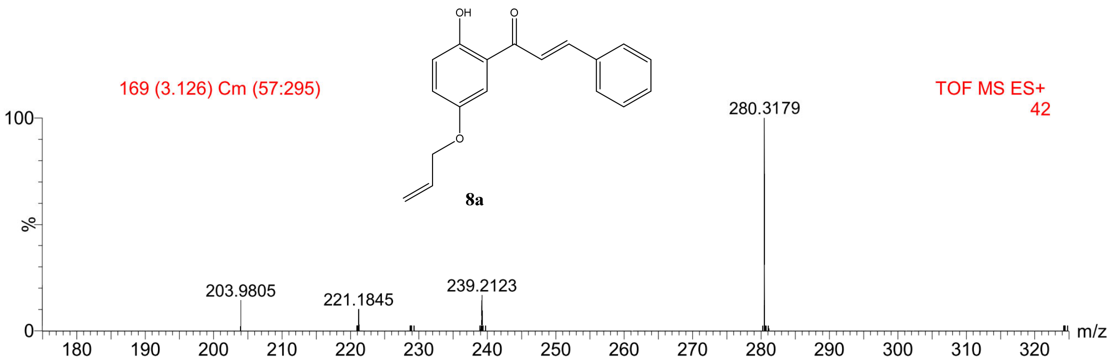

^{1}H NMR (400 MHz, CDCl₃) spectrum of compound 8b

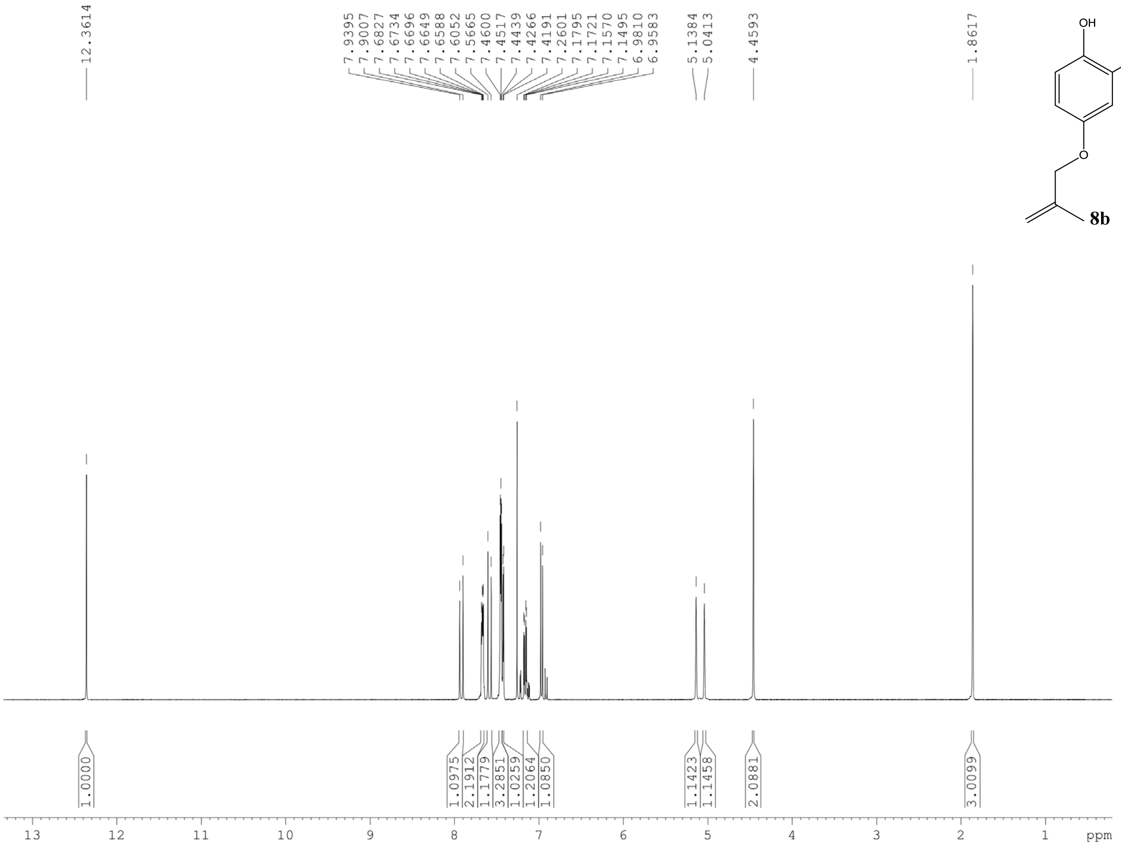

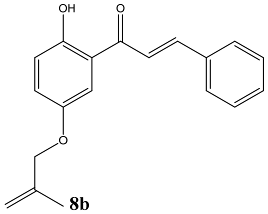

8b

^{13}C NMR (100 MHz, $CDCl_3$) spectrum of compound 8b

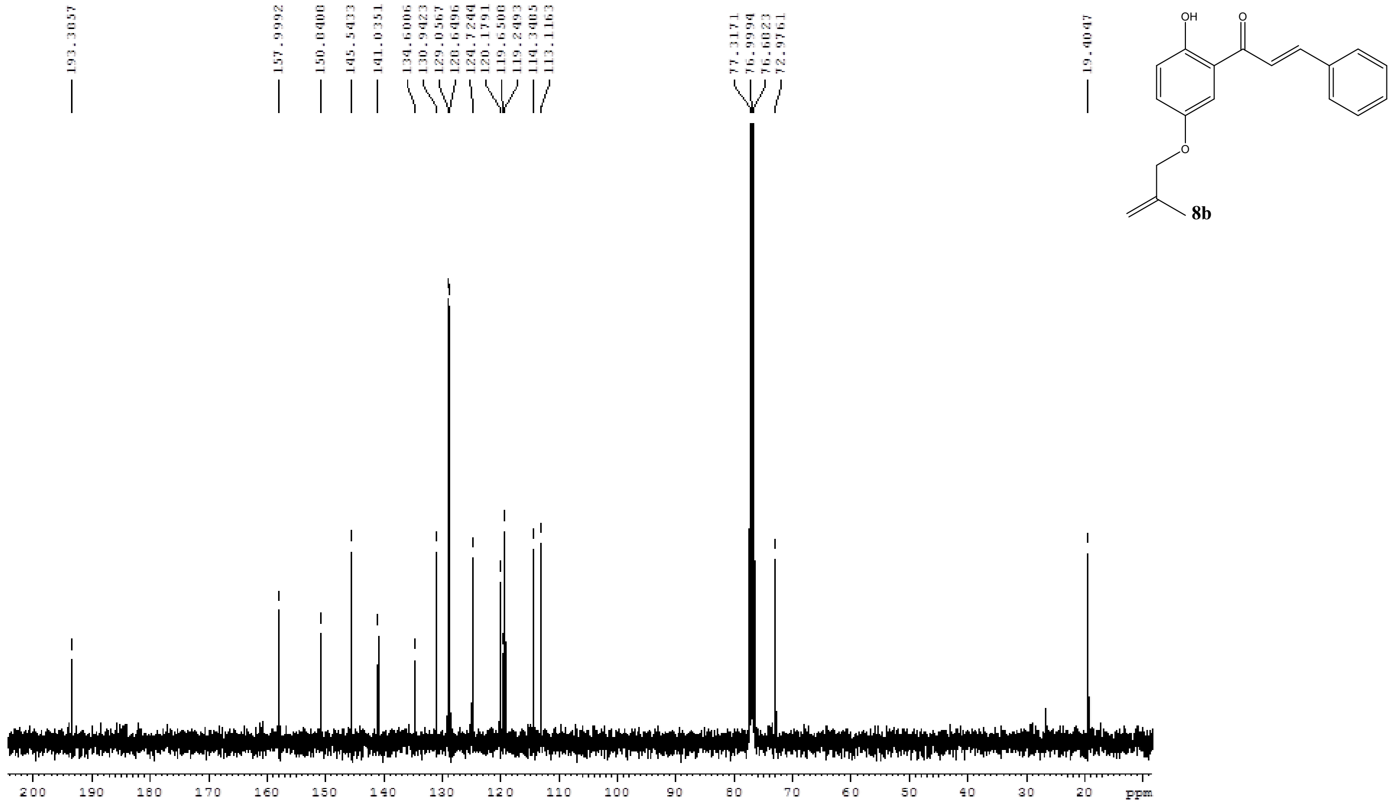

Mass of compound 8b

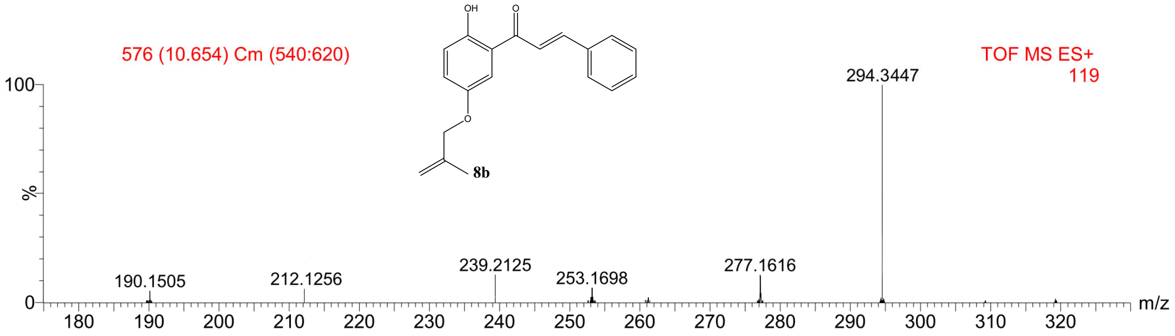

^{1}H NMR (400 MHz, $CDCl_3$) spectrum of compound 8c

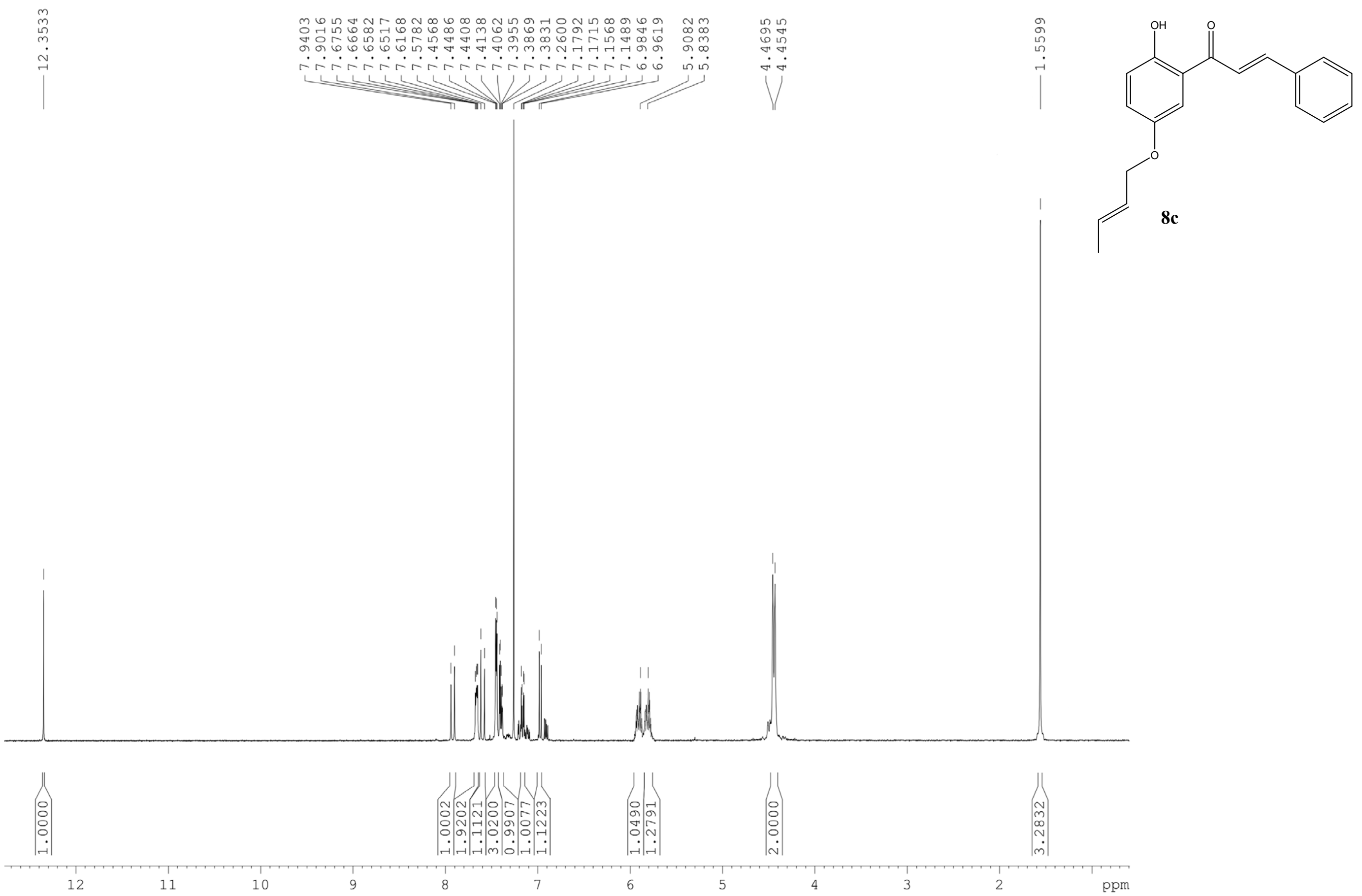

8c

^{13}C NMR (100 MHz, $CDCl_3$) spectrum of compound 8c

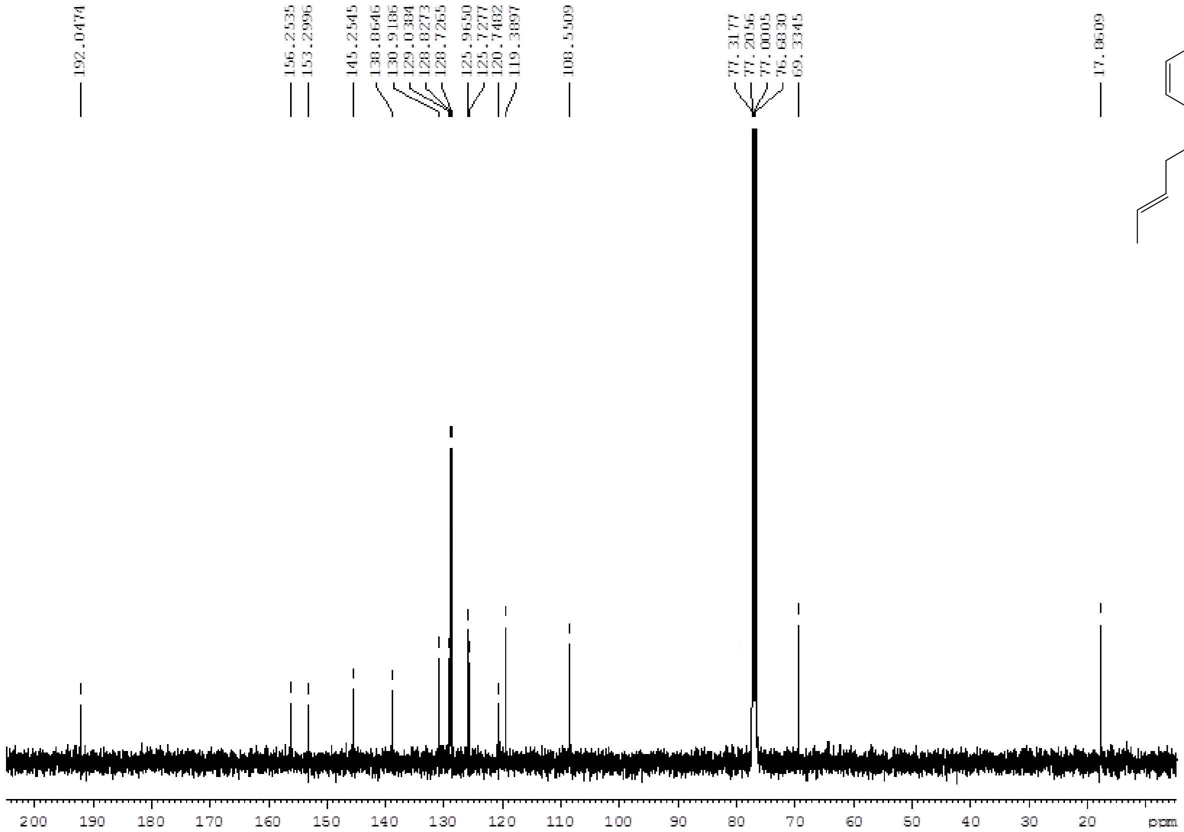

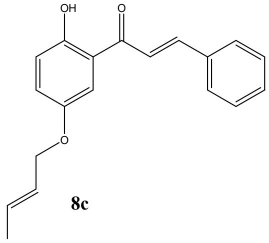

8c

Mass of compound 8c

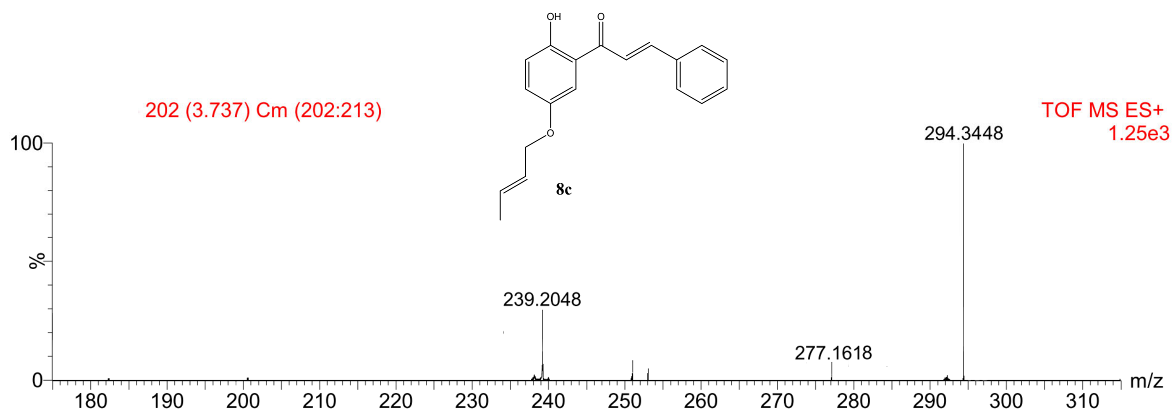

^{1}H NMR (400 MHz, CDCl3) spectrum of compound 8d

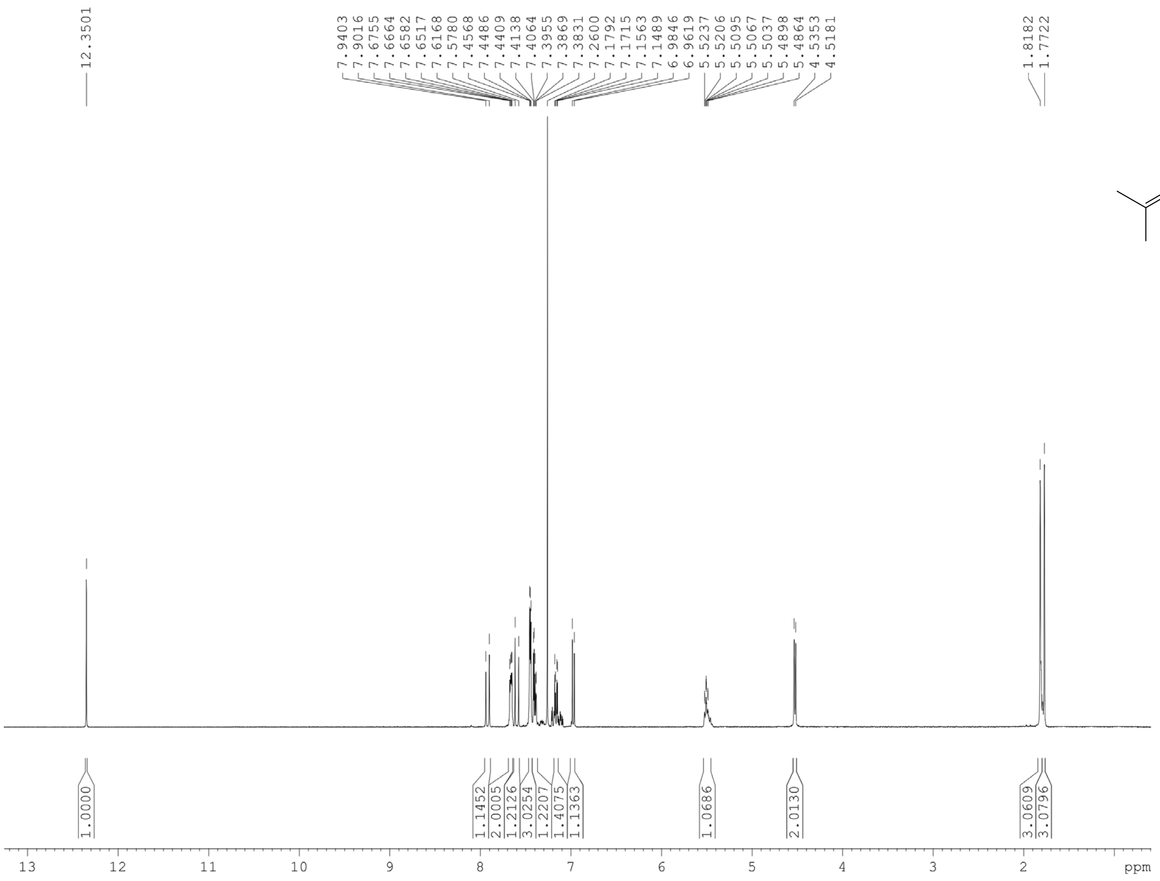

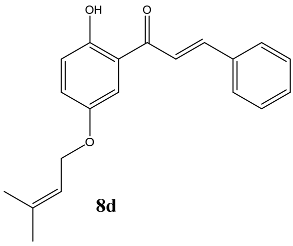

8d

^{13}C NMR (100 MHz, $CDCl_3$) spectrum of compound 8d

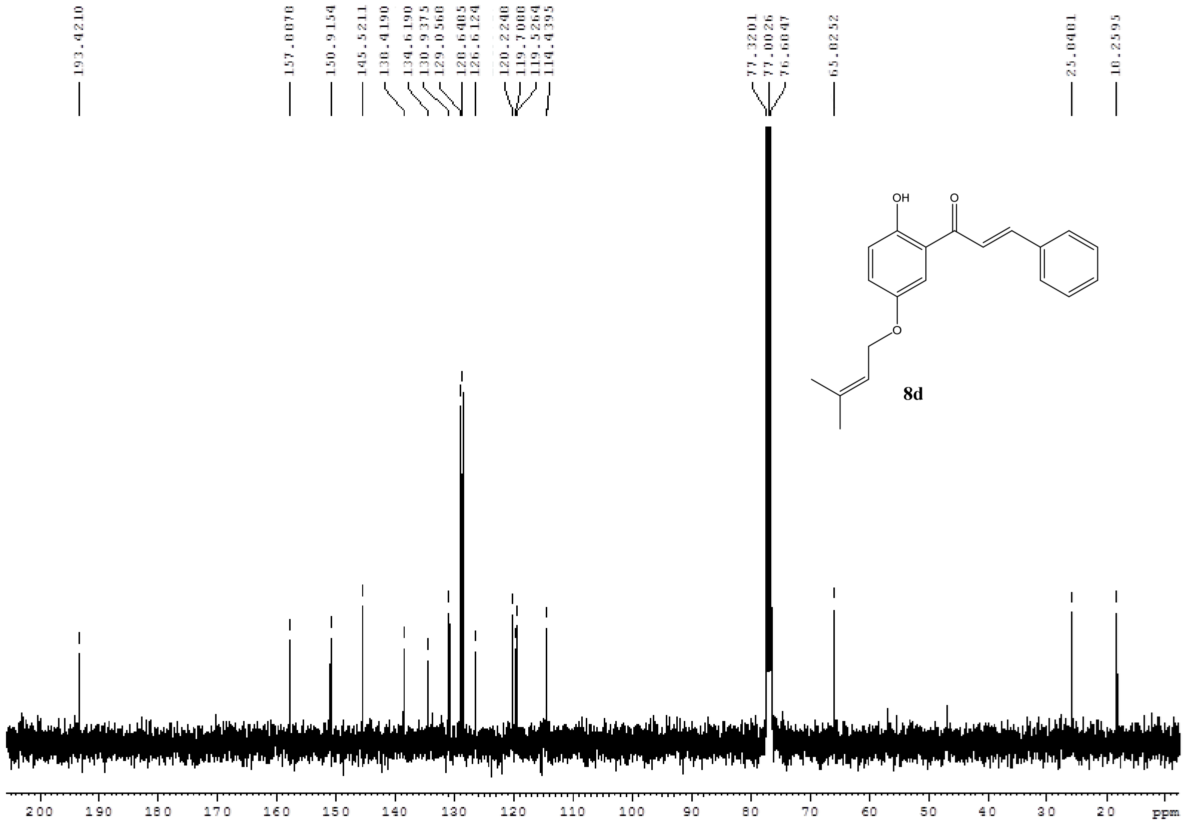

Mass of compound 8d

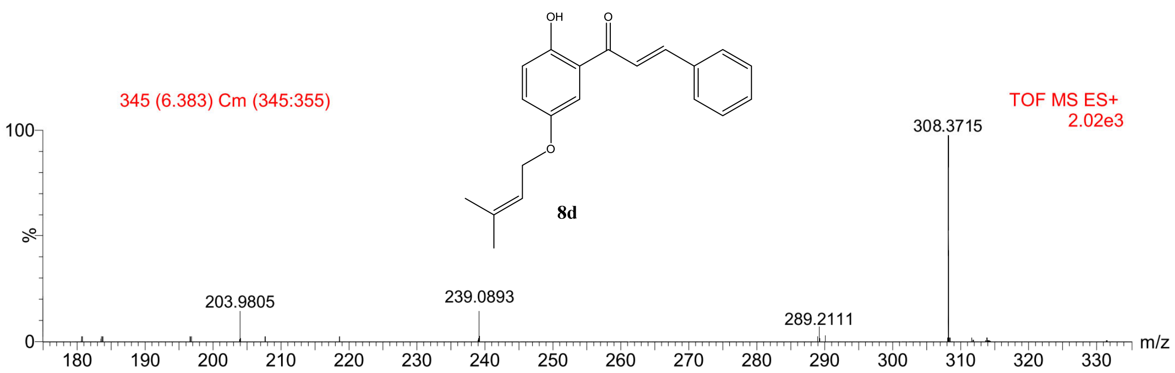

^{1}H NMR (400 MHz, $CDCl_3$) spectrum of compound 9a

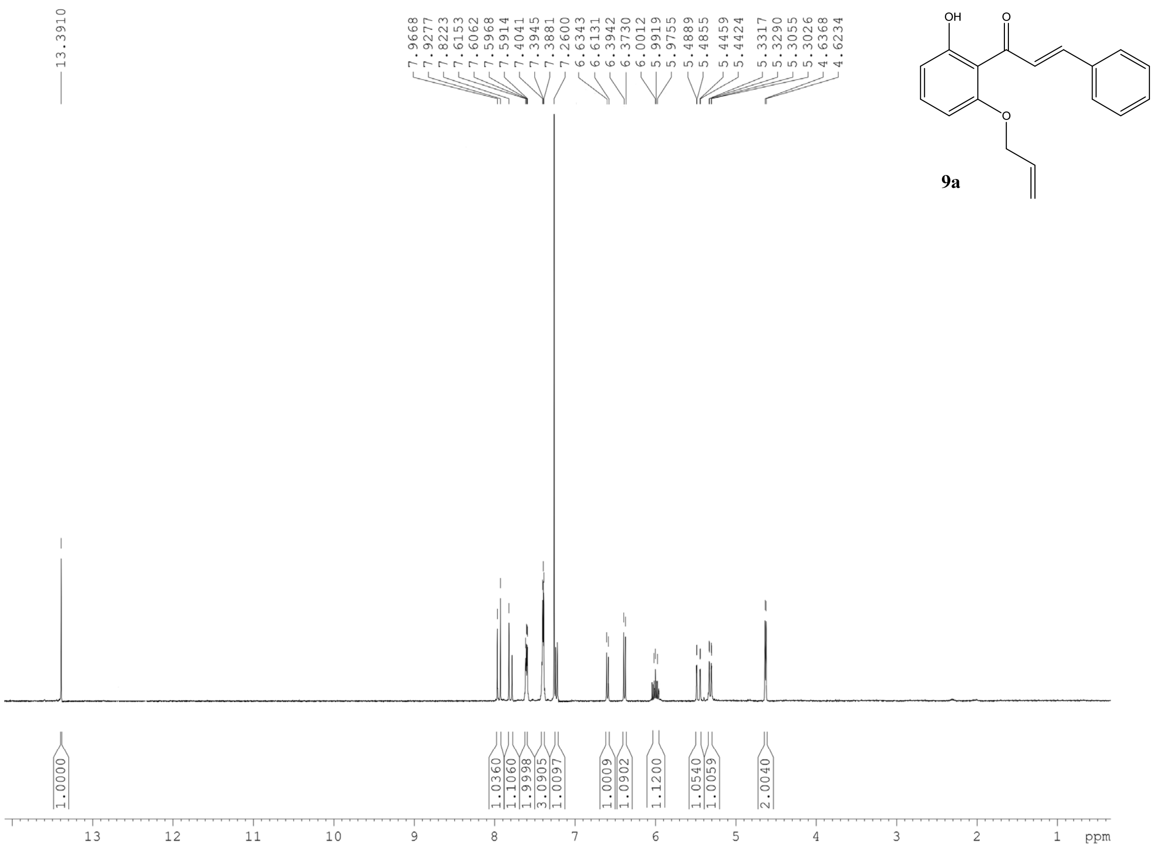

^{13}C NMR (100 MHz, $CDCl_3$) spectrum of compound 9a

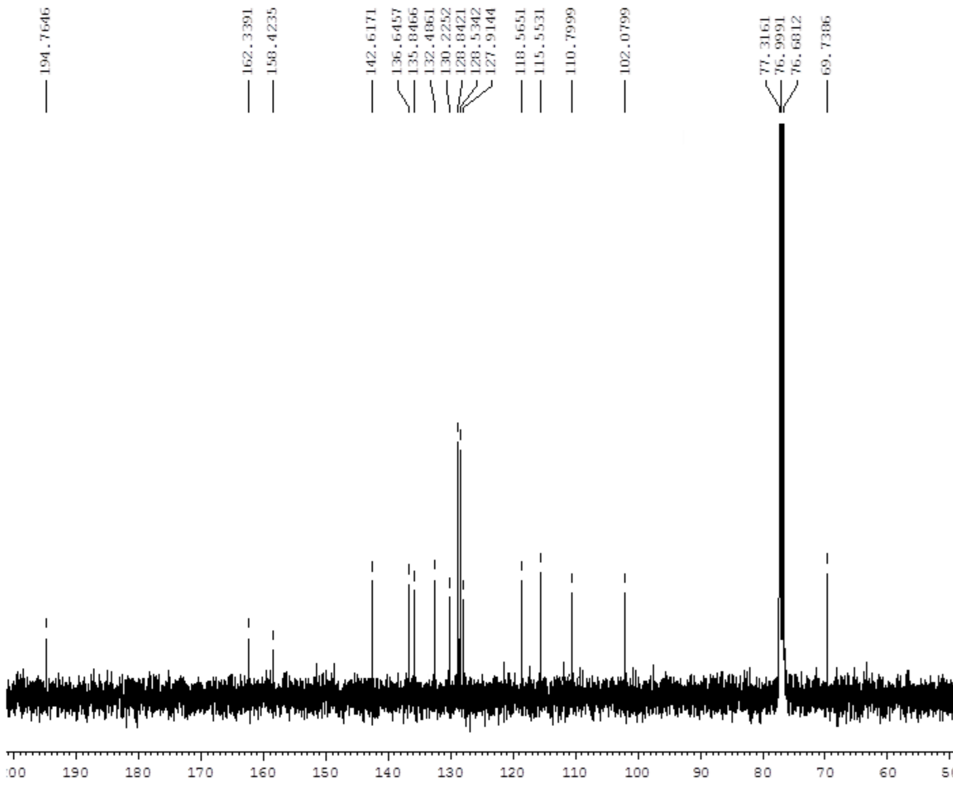

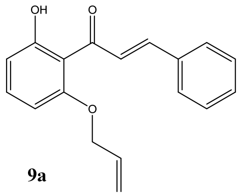

9a

Mass of compound 9a

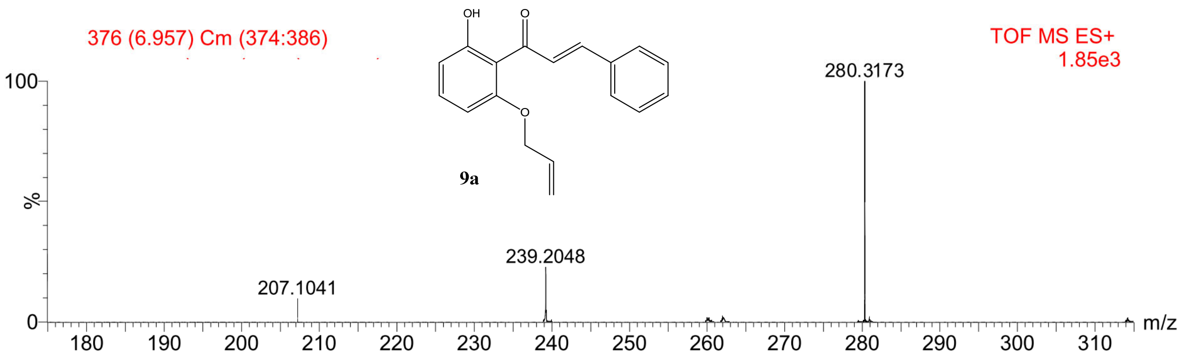

^{1}H NMR (400 MHz, CDCl3) spectrum of compound 9b

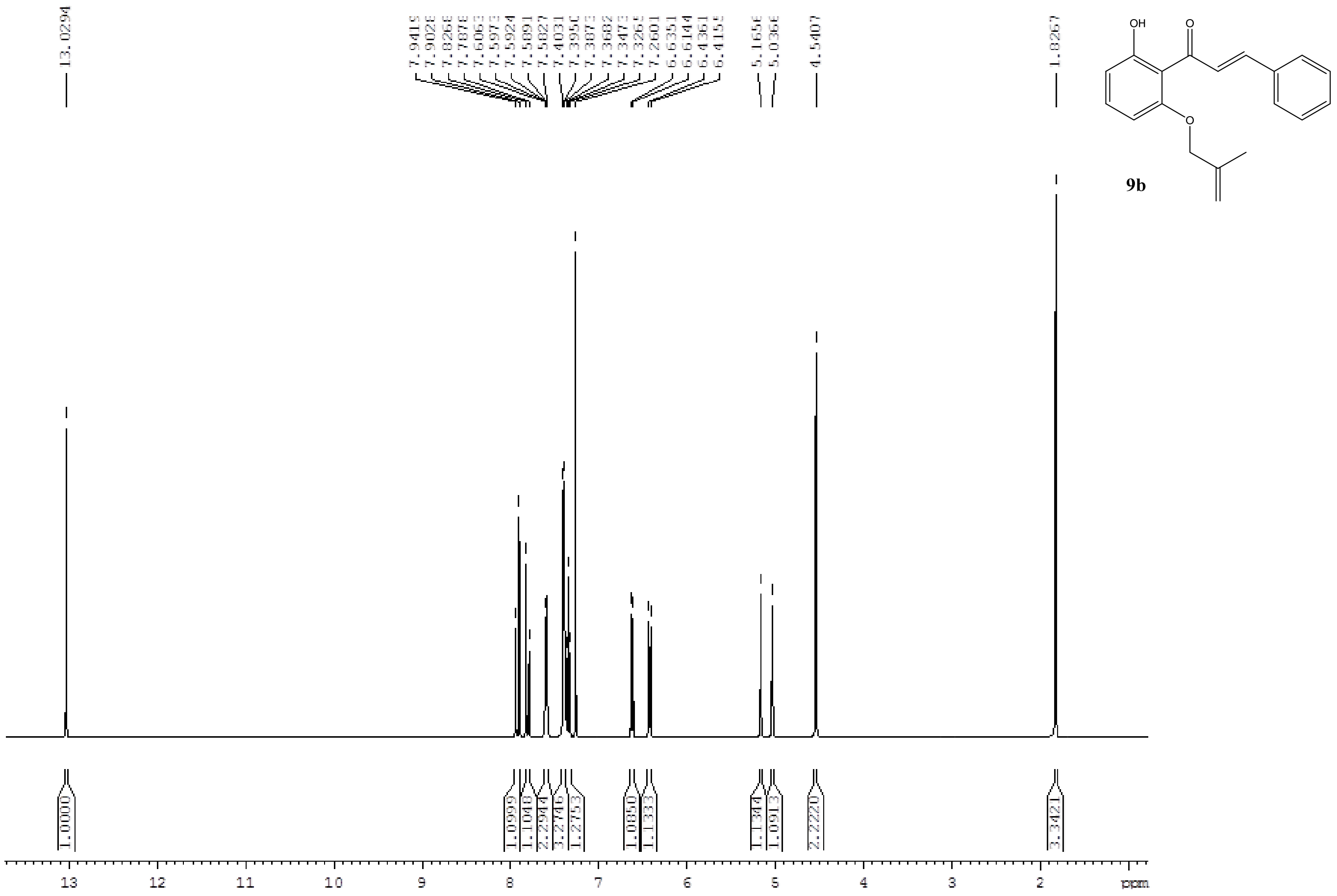

^{13}C NMR (100 MHz, $CDCl_3$) spectrum of compound 9b

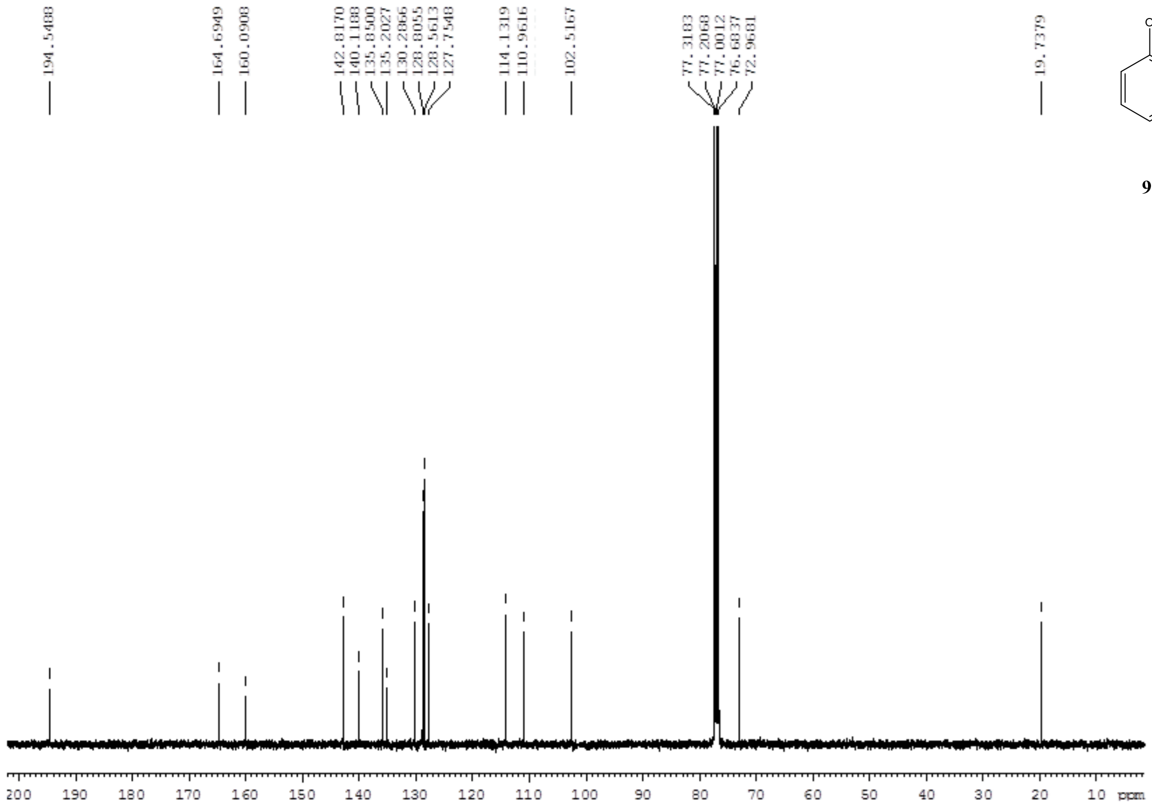

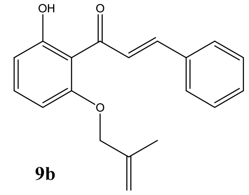

9b

Mass of compound 9b

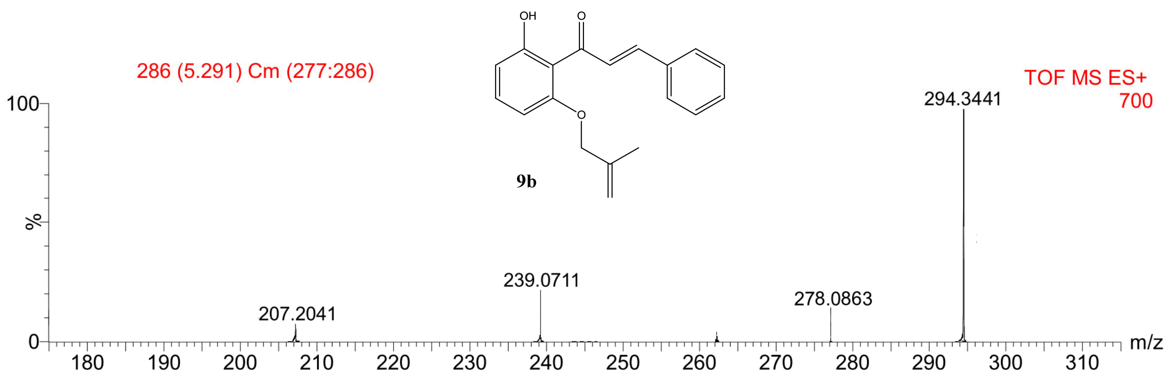

^{1}H NMR (400 MHz, $CDCl_3$) spectrum of compound 9c

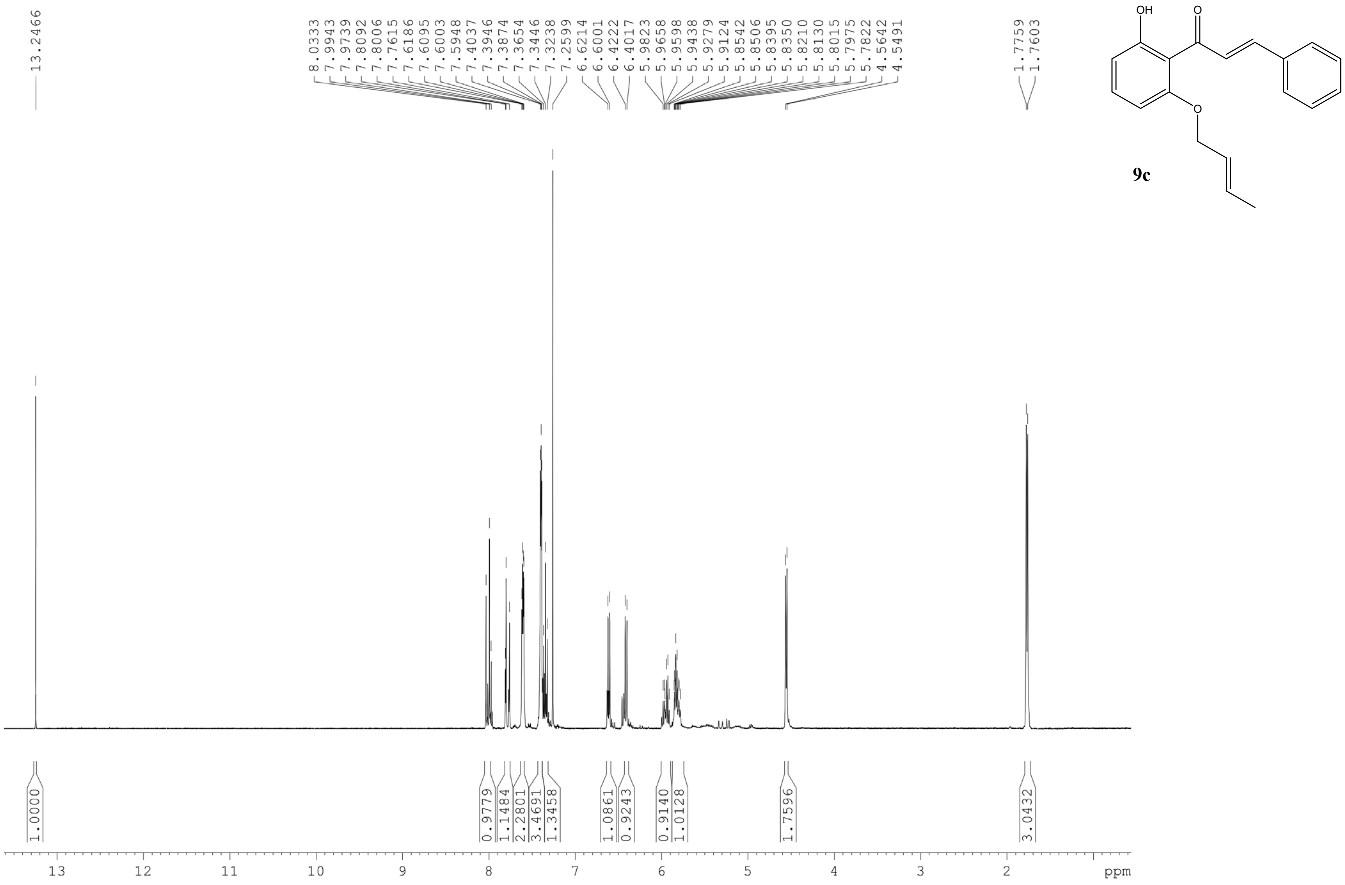

9c

^{13}C NMR (100 MHz, CDCl3) spectrum of compound 9c

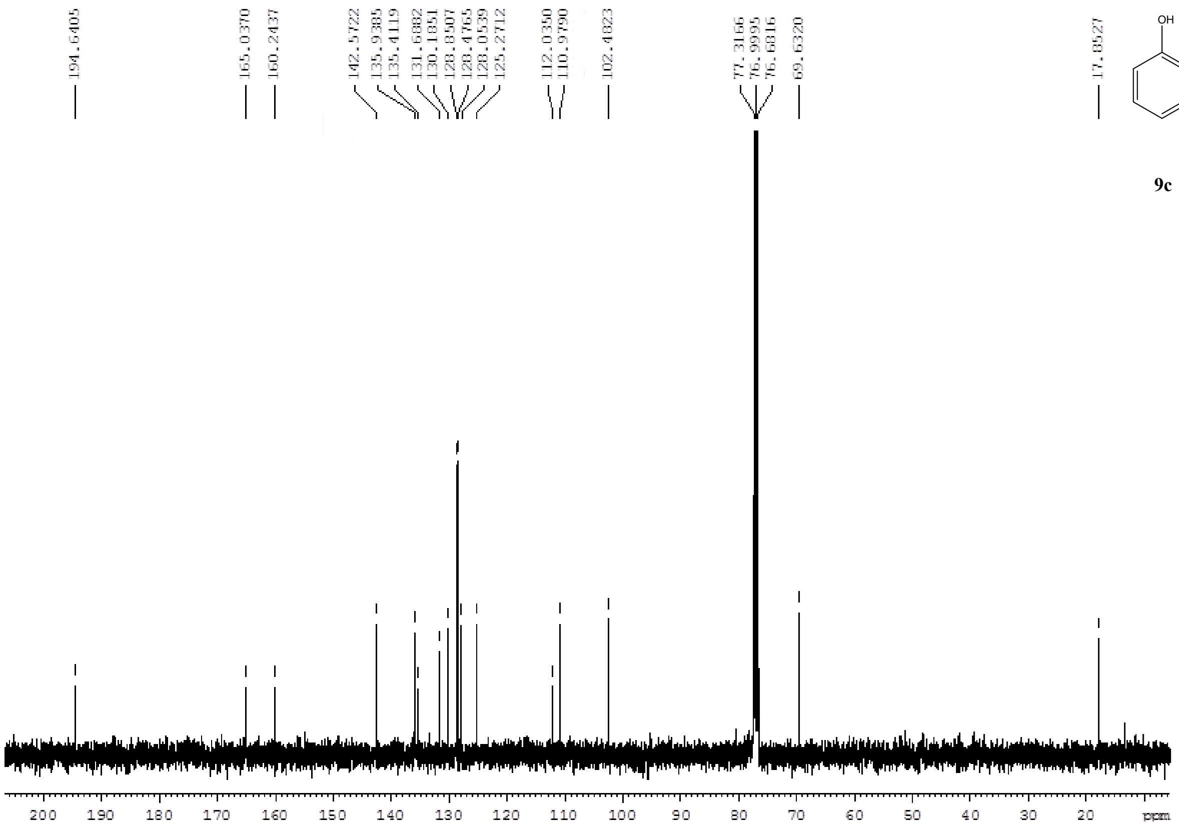

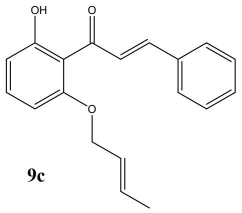

9c

Mass of compound 9c

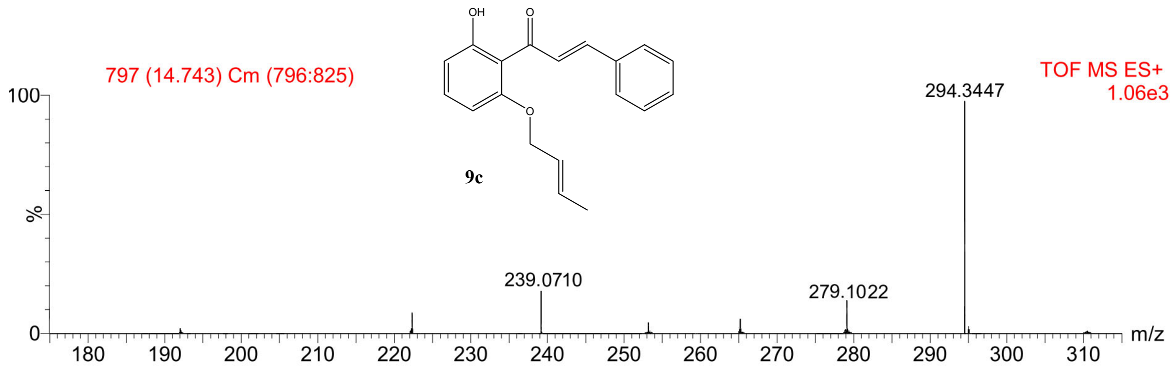

^{1}H NMR (400 MHz, CDCl3) spectrum of compound 9d

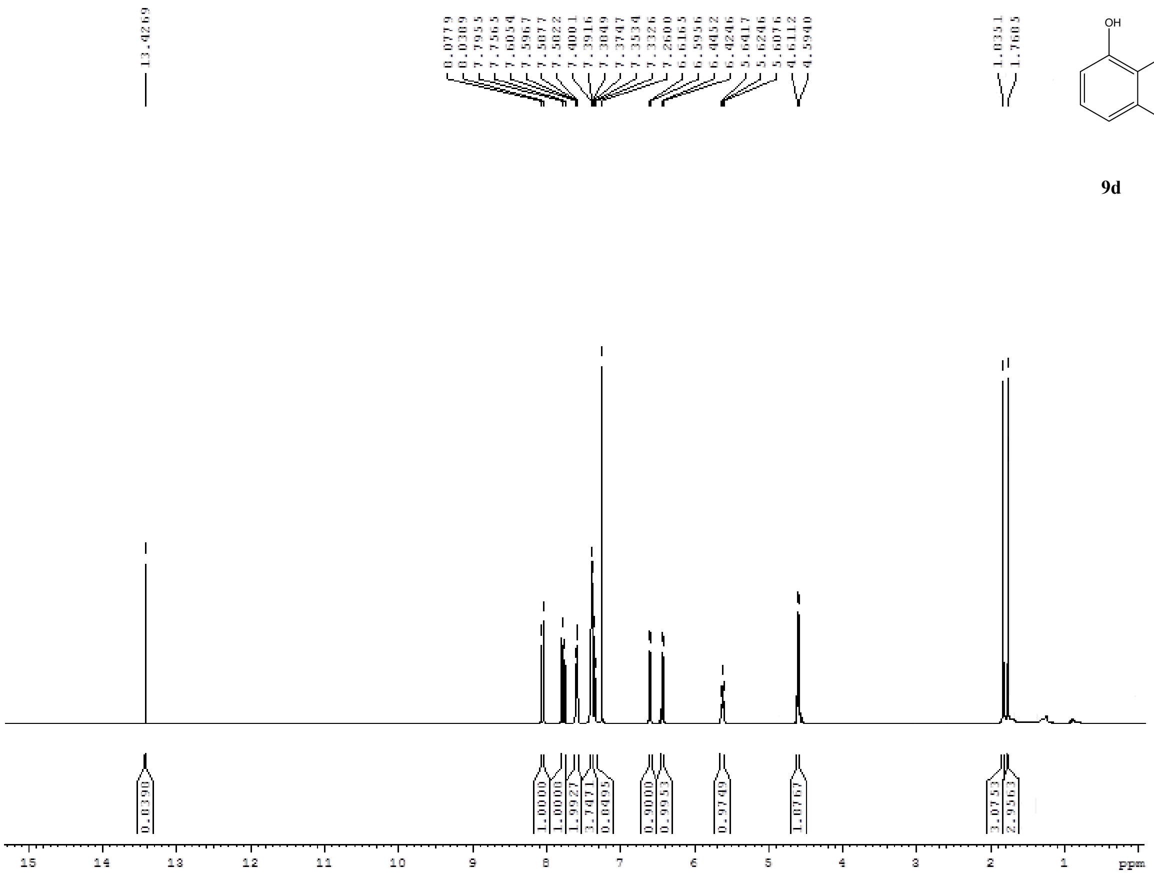

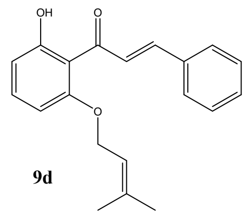

^{13}C NMR (100 MHz, $CDCl_3$) spectrum of compound 9d

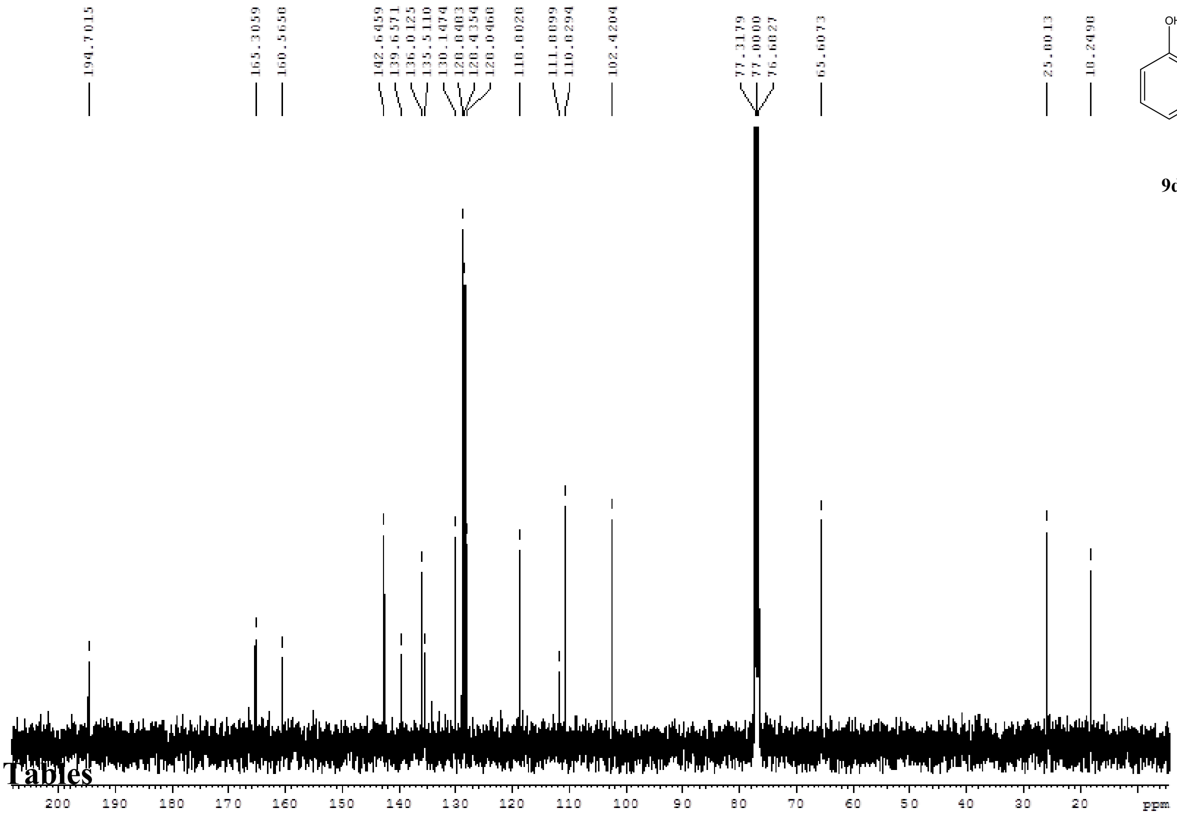

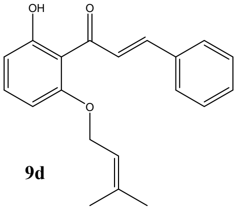

9d

Tables

Mass of compound 9d

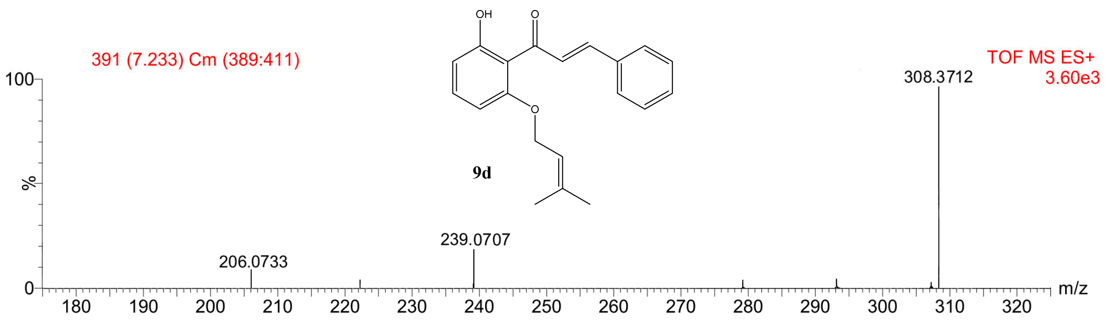

Table 1. Result of quantitative structure activity relationship in gas phase.

$pEC_{50} = 5.49(0.25) – 0.005(0.0008)MS + 0.464(0.46)C_{3'}$ N= 12; r= 0.925; r^2= 0.856; SD= 0.032; F= 26.8; q^2= 0.832					
**Compound**	**Descriptors**		**pEC_{50}**		**Residual**
	**MS**	**$C_{3'}$**	**Experimental**	**Calculated**	
**7a**	274.488	-0.436	3.804	3.783	0.021
**7b**	292.74	-0.439	3.643	3.682	-0.039
**7c**	294.889	-0.464	3.640	3.658	-0.018
**7d**	310.99	-0.397	3.609	3.601	0.008
**8a**	274.385	-0.254	3.930	3.869	0.062
**8b**	292.611	-0.23	3.741	3.780	-0.039
**8c**	294.79	-0.249	3.742	3.759	-0.017
**8d**	310.9	-0.246	3.663	3.672	-0.009
**9a**	273.381	-0.352	3.804	3.829	-0.024
**9b**	289.812	-0.379	3.707	3.726	-0.019
**9c**	293.786	-0.365	3.724	3.710	0.013
**9d**	310.067	-0.39	3.670	3.609	0.061

Table 2. Result of quantitative structure activity relationship in condensed phase.

$pEC_{50} = 5.51(0.24) – 0.006(0.0008)MS + 0.472(0.13)C_{3'}$ N= 12; r= 0.934; r^2= 0.871; SD= 0.029; F= 30.5; q^2= 0.852					
**Compound**	**Descriptors**		**pEC_{50}**		**Residual**
	**MS**	**$C_{3'}$**	**Experimental**	**Calculated**	
**7a**	274.488	-0.448	3.804	3.781	0.023
**7b**	292.74	-0.459	3.643	3.675	-0.032
**7c**	294.889	-0.471	3.64	3.657	-0.017
**7d**	310.99	-0.395	3.609	3.604	0.005
**8a**	274.385	-0.251	3.930	3.875	0.056
**8b**	292.611	-0.242	3.741	3.778	-0.038
**8c**	294.79	-0.251	3.742	3.762	-0.020
**8d**	310.9	-0.256	3.663	3.670	-0.007
**9a**	273.381	-0.364	3.804	3.827	-0.023
**9b**	289.812	-0.382	3.707	3.728	-0.021
**9c**	293.786	-0.374	3.724	3.709	0.014
**9d**	310.067	-0.392	3.670	3.611	0.06

Table 3. Proposal compounds based in QSAR analysis based in **7a** core.

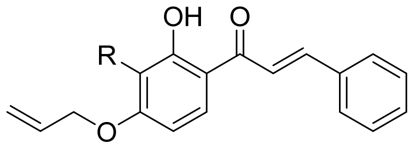

Compound	R	MS	C3′	pIC50 Calc
**Test_1**	H	274.488	-0.448	3.781
**Test_2**	F	279.027	-0.014	3.829
**Test_3**	Cl	288.424	-0.451	3.57
**Test_4**	Br	293.425	-0.651	3.447
**Test_5**	CN	289.785	-0.548	3.517
**Test_6**	CHO	290.634	-0.463	3.551
**Test_7**	NO_2	293.698	-0.328	3.596
**Test_8**	Me	292.08	-0.135	3.695
**Test_9**	OH	282.496	0.023	3.826
**Test_10**	Ome	300.033	0.066	3.74
**Test_11**	NH_2	284.616	-0.088	3.761
**Test_12**	NMe_2	316.854	-0.043	3.589

Table 4. Proposal compounds based in QSAR analysis based in **8a** core.

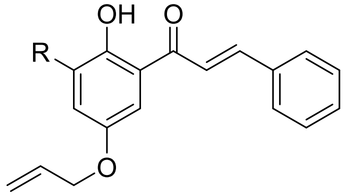

Compound	R	MS	C3′	pIC50 Calc
**Test_1**	H	274.385	-0.251	3.745
**Test_2**	F	277.802	0.289	3.98
**Test_3**	Cl	288.466	-0.112	3.726
**Test_4**	Br	293.738	-0.271	3.62
**Test_5**	CN	289.272	-0.214	3.673
**Test_6**	CHO	290.77	0.096	3.811
**Test_7**	NO_2	293.906	-0.171	3.666
**Test_8**	Me	292.317	0.184	3.843
**Test_9**	OH	281.185	0.278	3.954
**Test_10**	OMe	301.735	0.138	3.765
**Test_11**	NH_2	284.899	0.23	3.909
**Test_12**	NMe_2	320.063	0.154	3.662

Table 5. Proposal compounds based in QSAR analysis based in **9a** core.

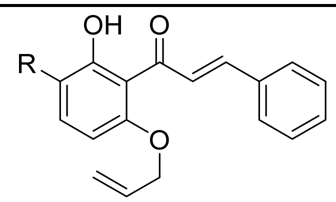

Compound	R	MS	C_3	pIC_{50} Calc
**Test_1**	H	273.381	-0.364	3.827
**Test_2**	F	273.92	0.068	3.898
**Test_3**	Cl	283.853	-0.264	3.684
**Test_4**	Br	288.885	-0.466	3.56
**Test_5**	CN	285.025	-0.330	3.647
**Test_6**	CHO	286.103	-0.309	3.65
**Test_7**	NO_2	292.96	-0.258	3.633
**Test_8**	Me	290.964	0.028	3.777
**Test_9**	OH	278.041	0.148	3.910
**Test_10**	Ome	297.139	0.155	3.799
**Test_11**	NH_2	280.34	0.311	3.972
**Test_12**	NMe_2	317.798	0.100	3.650
